# Supplementary material for: Differing taxonomic responses of mosquito vectors to anthropogenic land-use change in Latin America and the Caribbean
Source: PLoS Negl Trop Dis. 2023 Jul 14;17(7):e0011450. doi: 10.1371/journal.pntd.0011450 (PMC10348580; doi:10.1371/journal.pntd.0011450)
Supplement: S1 Text — Systematic search strategy used to find and extract mosquito abundance data, including inclusion and exclusion criteria. (DOCX) [file pntd.0011450.s001.docx]

**Systematic data search strategy**

A systematic review approach was employed to find and extract relevant abundance data on mosquito biodiversity across multiple land-use types in LAC. We focused on mosquitoes of the genus *Anopheles* and *Aedes* due to the high burden of disease these vectors transmit in the region and the large diversity of mosquito genera. A systematic literature search was performed on 5^th^ July 2021 across three databases (Medline, Scopus, and Web of Science; S1 Fig). Country- and region-specific search terms were used to capture studies within geographical boundaries of LAC, alongside a combination of mosquito-specific e.g., *Aedes* and *Anopheles*, and land use specific search terms e.g., deforestation and agriculture (complete search term details are provided in S1 Table). Results from each database search were combined and duplicates removed. Studies were first screened by title and abstract to remove studies that were out of scope, such as epidemiological interventions and laboratory-based analyses. The full texts of potentially relevant studies were then reviewed. To qualify for inclusion, studies had to contain available abundance data (either in the main text or supplementary) for one or more *Anopheles* or *Aedes* mosquito vector species and include samples across multiple land-use types. If a study included data on at least one mosquito vector (judged if there was evidence from the published literature linking the mosquito to at least one human disease), then all *Aedes* and *Anopheles* data from that study were collected. Studies were excluded if there was an insufficient description of the land use or land cover of the sampled site, or if the description was too crude. For example, studies describing sites as ‘wild’ or ‘peridomestic’ were excluded. Studies were also excluded if only one land-use type was sampled and if the sampling methodology was not adequately described, for example if the study did not include sampling effort.
