# Supplementary material for: Differing taxonomic responses of mosquito vectors to anthropogenic land-use change in Latin America and the Caribbean
Source: PLoS Negl Trop Dis. 2023 Jul 14;17(7):e0011450. doi: 10.1371/journal.pntd.0011450 (PMC10348580; doi:10.1371/journal.pntd.0011450)
Supplement: S1 Table — Mosquito, geographical and land use specific terms used to systematically search three databases (Medline, Scopus and Web of Science) for Aedes and Anopheles mosquito abundance records across multiple land-use types in Latin America and the Caribbean. * denotes wildcard terms. (DOCX) [file pntd.0011450.s002.docx]

| **Mosquito search terms** |
| --- |
| mosquito* OR anophel* or aedes |
| **Geographical search terms** |
| (south* AND america*) OR amazon* OR brazil* OR bolivia* OR colombia* OR surinam* OR guiana* OR venezuela* OR ecuador* OR peru* OR guyana* OR chile* OR argentin* OR uruguay* OR paraguay* OR america* OR (central AND america*) OR (latin AND america*) OR caribbean OR anguilla* OR antigua* OR barbuda OR (antigua* AND barbuda*) OR aruba* OR bahama* OR baham* OR barbados* OR barbadian* OR belize* OR bermud* OR (british AND virgin AND island*) OR cayman OR (cayman AND island*) OR (costa AND rica*) OR cuba* OR curacao* OR dominica* OR (dominica* AND republic) OR (el AND salvador*) OR grenad* OR guadeloup* OR guatemala* OR haiti* OR hondura* OR jamaica* OR martiniqu* OR mexic* OR montserrat* OR antill* OR nicaragua* OR panama* OR (puerto AND ric*) OR (saint AND kitts AND nevis) OR (saint AND kitts) OR (saint AND lucia*) OR (saint AND vincent) OR (saint AND vincent AND grenadine*) OR (saint AND martin) OR (sint AND maarten) OR trinidad* OR (trinidad AND tobago) OR tobago* OR (turks AND caicos)) |
| **Land use search terms** |
| land* OR urban* OR deforest* OR logg* OR intensification OR manag* OR unmanage* OR felling OR plantation OR habitat* OR forest* OR mining OR mine* OR clear* OR degrad* OR develop* OR agricultur* OR landscape* OR crop* OR farm* OR canal* OR dams OR dam OR pond* |
