# Supplementary material for: Differing taxonomic responses of mosquito vectors to anthropogenic land-use change in Latin America and the Caribbean
Source: PLoS Negl Trop Dis. 2023 Jul 14;17(7):e0011450. doi: 10.1371/journal.pntd.0011450 (PMC10348580; doi:10.1371/journal.pntd.0011450)
Supplement: S2 Table — Site-level information extracted from each included study to formulate a dataset of mosquito vector biodiversity over different land-use types. The nested structure of the dataset (study number, site number, study block and study sample) followed that of the PREDICTS database (37). (DOCX) [file pntd.0011450.s003.docx]

| **Name** | **Description** |
| --- | --- |
| Collection | Sample collection indoor or outdoor |
| Country | Country sample was performed |
| Development stage | Sampled mosquito development stage (pupae, larvae, immature, adult) |
| Ecoregion | Assigned ecoregion according to the 14 WWF global terrestrial ecoregions (WWF, 2021) |
| Genus | Mosquito genus |
| Site description | Detailed description of sampled site, according to study |
| Land use | Predominant land-use type (primary vegetation, secondary vegetation, managed or urban) |
| Land-use intensity | Predominant intensity of land use (minimal or substantial) |
| Lat | Latitude |
| Lon | Longitude |
| Measurement | Value of species-level abundance measurement |
| Metric | Metric of abundance measurement (e.g. number of individuals or larval density) |
| Reference | Study reference |
| Sample daytime | Time of day when sample was taken |
| Sample start | Start date of sample |
| Sample end | End date of sample |
| Sample month | Month of sampling |
| Sample season | Climatic season of sampling (wet or dry) |
| Sampling effort | Value of sampling effort |
| Sampling effort unit | Unit of sampling effort (e.g. trap days or man hours) |
| Sampling method | Method of sampling (e.g. light trap, oviposition trap or human landing catch) |
| Site number | Assigned site number within study |
| Species study name | Species name according to study |
| Study block | Spatial arrangement of site within a study |
| Study number | Assigned study number |
| Study sample | Sample within a study with consistent sampling methodology |
