# Supplementary material for: Differing taxonomic responses of mosquito vectors to anthropogenic land-use change in Latin America and the Caribbean
Source: PLoS Negl Trop Dis. 2023 Jul 14;17(7):e0011450. doi: 10.1371/journal.pntd.0011450 (PMC10348580; doi:10.1371/journal.pntd.0011450)
Supplement: S3 Table — Description of land-use types used to classify sample sites in the dataset. Categories were adapted following Hudson et al. (37) and Gibb et al. (8). (DOCX) [file pntd.0011450.s004.docx]

| **Land-use category** | **Description** |
| --- | --- |
| Primary vegetation | Natural vegetation with no evidence of previous destruction |
| Secondary vegetation | Vegetation that is recovering after removal of the natural vegetation |
| Managed | Agricultural land used for plantations (cultivation of woody crops, such as oil-palm, rubber, fruit, coffee, or timber), cropland (cultivation of herbaceous crops, including fodder for livestock), and pasture (livestock grazing) |
| Urban | Areas of human habitation and buildings, from small green spaces, through to villages and cities |
