# Supplementary material for: Differing taxonomic responses of mosquito vectors to anthropogenic land-use change in Latin America and the Caribbean
Source: PLoS Negl Trop Dis. 2023 Jul 14;17(7):e0011450. doi: 10.1371/journal.pntd.0011450 (PMC10348580; doi:10.1371/journal.pntd.0011450)
Supplement: S4 Table — Site-level distribution and number of abundance records per land-use category. Use intensity for managed and urban land-use types were aggregated due to low data representation. (DOCX) [file pntd.0011450.s005.docx]

| **Land-use intensity** | **Number of sites** | **Number of records** |
| --- | --- | --- |
| Primary vegetation |  |  |
| Minimal | 224 | 2,388 |
| Substantial | 68 | 1,447 |
| Secondary vegetation |  |  |
| Combined | 57 | 1,325 |
| Managed |  |  |
| Combined | 98 | 1,420 |
| Urban |  |  |
| Combined | 195 | 3,664 |
