# Supplementary material for: Differing taxonomic responses of mosquito vectors to anthropogenic land-use change in Latin America and the Caribbean
Source: PLoS Negl Trop Dis. 2023 Jul 14;17(7):e0011450. doi: 10.1371/journal.pntd.0011450 (PMC10348580; doi:10.1371/journal.pntd.0011450)
Supplement: S5 Table — Summary of components of total, Aedes and Anopheles abundance and species richness models. The number of sites and site-level records in each model is shown. (DOCX) [file pntd.0011450.s006.docx]

| **Model** | **Response** | **Random effects** | **Number of sites** | **Number of site-level records** |
| --- | --- | --- | --- | --- |
| Total abundance | Total log(adjusted abundance)+1 | study, site, species, study sample, ecoregion | 632 | 4,582 |
| *Aedes* abundance | log(adjusted abundance)+1 | study, site, species, study sample, ecoregion | 379 | 2,118 |
| *Anopheles* abundance | log(adjusted abundance)+1 | study, site, species, study sample, ecoregion | 495 | 2,464 |
| Total species richness | Total (*Aedes* and *Anopheles*) species richness | study, site, study sample, ecoregion | 434 | 656 |
| *Aedes* species richness | *Aedes* species richness | study, site, study sample, ecoregion | 238 | 330 |
| *Anopheles* species richness | *Anopheles* species richness | study, site, study sample, ecoregion | 291 | 433 |
