# Supplementary material for: Differing taxonomic responses of mosquito vectors to anthropogenic land-use change in Latin America and the Caribbean
Source: PLoS Negl Trop Dis. 2023 Jul 14;17(7):e0011450. doi: 10.1371/journal.pntd.0011450 (PMC10348580; doi:10.1371/journal.pntd.0011450)
Supplement: S6 Table — Site-level distribution and number of site-level abundance records per Aedes and Anopheles species with greatest representation in the dataset. (DOCX) [file pntd.0011450.s007.docx]

| **Species** | **Number of sites** | **Number of site-level records** | **Disease(s)** |
| --- | --- | --- | --- |
| *Aedes aegypti* | 233 | 422 | Dengue, zika, chikungunya, Mayaro virus, yellow fever |
| Primary vegetation – minimal | 58 | 104 |  |
| Primary vegetation – substantial | 18 | 33 |  |
| Secondary vegetation | 22 | 27 |  |
| Managed | 10 | 19 |  |
| Urban | 125 | 239 |  |
| *Aedes albopictus* | 248 | 519 | Dengue, zika, chikungunya, yellow fever |
| Primary vegetation – minimal | 73 | 153 |  |
| Primary vegetation – substantial | 22 | 50 |  |
| Secondary vegetation | 29 | 40 |  |
| Managed | 6 | 9 |  |
| Urban | 128 | 267 |  |
| *Aedes scapularis* | 230 | 395 | Chikungunya, yellow fever |
| Primary vegetation – minimal | 74 | 146 |  |
| Primary vegetation – substantial | 23 | 29 |  |
| Secondary vegetation | 24 | 30 |  |
| Managed | 19 | 24 |  |
| Urban | 90 | 166 |  |
| *Aedes serratus* | 206 | 363 | Yellow fever, chikungunya |
| Primary vegetation – minimal | 71 | 140 |  |
| Primary vegetation – substantial | 15 | 20 |  |
| Secondary vegetation | 18 | 23 |  |
| Managed | 14 | 16 |  |
| Urban | 88 | 164 |  |
| *Anopheles albimanus* | 22 | 30 | Malaria |
| Primary vegetation – minimal | 2 | 4 |  |
| Primary vegetation – substantial | 8 | 8 |  |
| Secondary vegetation | 5 | 7 |  |
| Managed | 4 | 8 |  |
| Urban | 3 | 3 |  |
| *Anopheles albitarsis* | 68 | 129 | Malaria |
| Primary vegetation – minimal | 22 | 39 |  |
| Primary vegetation – substantial | 13 | 23 |  |
| Secondary vegetation | 8 | 15 |  |
| Managed | 17 | 39 |  |
| Urban | 8 | 13 |  |
| *Anopheles darlingi* | 170 | 354 | Malaria |
| Primary vegetation – minimal | 88 | 149 |  |
| Primary vegetation – substantial | 17 | 28 |  |
| Secondary vegetation | 10 | 24 |  |
| Managed | 37 | 113 |  |
| Urban | 18 | 40 |  |
| *Anopheles nuneztovari* | 55 | 111 | Malaria |
| Primary vegetation – minimal | 15 | 18 |  |
| Primary vegetation – substantial | 11 | 12 |  |
| Secondary vegetation | 9 | 20 |  |
| Managed | 11 | 34 |  |
| Urban | 9 | 27 |  |
