# Supplementary material for: Differing taxonomic responses of mosquito vectors to anthropogenic land-use change in Latin America and the Caribbean
Source: PLoS Negl Trop Dis. 2023 Jul 14;17(7):e0011450. doi: 10.1371/journal.pntd.0011450 (PMC10348580; doi:10.1371/journal.pntd.0011450)
Supplement: S7 Table — Deviance information criterion (DIC) and Watanabe-Akaike information criterion (WAIC) for models of total, Aedes and Anopheles species richness with the addition of random effects structures. Each random effect was added iteratively to assess model performance. (DOCX) [file pntd.0011450.s008.docx]

| **Species** | **Random effects** | **DIC** | **WAIC** |
| --- | --- | --- | --- |
| Total | Study number + site number | 2347.31 | 2332.30 |
|  | … + study block | 2335.50 | 2315.08 |
|  | … + study sample | 2332.41 | 2306.23 |
|  | … + ecoregion | 2335.72 | 2315.47 |
| *Aedes* | Study number + site number | 976.69 | 961.14 |
|  | … + study block | 976.79 | 961.14 |
|  | … + study sample | 977.02 | 961.21 |
|  | … + ecoregion | 976.82 | 961.15 |
| *Anopheles* | Study number + site number | 1633.07 | 1617.43 |
|  | … + study block | 1623.78 | 1605.60 |
|  | … + study sample | 1623.71 | 1605.68 |
|  | … + ecoregion | 1623.76 | 1605.53 |
