# Supplementary material for: Differing taxonomic responses of mosquito vectors to anthropogenic land-use change in Latin America and the Caribbean
Source: PLoS Negl Trop Dis. 2023 Jul 14;17(7):e0011450. doi: 10.1371/journal.pntd.0011450 (PMC10348580; doi:10.1371/journal.pntd.0011450)
Supplement: S8 Table — Deviance information criterion (DIC) and Watanabe-Akaike information criterion (WAIC) for models of total, Aedes and Anopheles abundance with the addition of random effects structures. Each random effect was added iteratively to assess model performance. (DOCX) [file pntd.0011450.s009.docx]

| **Species** | **Random effects** | **DIC** | **WAIC** |
| --- | --- | --- | --- |
| Total | Study number + site number | 12408.30 | 12469.47 |
|  | … + study block | 12395.84 | 12472.46 |
|  | … + study sample | 12397.27 | 12468.61 |
|  | … + species | 11888.01 | 12007.19 |
|  | … + ecoregion | 11859.88 | 11976.07 |
| *Aedes* | Study number + site number | 6141.58 | 6196.44 |
|  | … + study block | 6121.02 | 6192.49 |
|  | … + study sample | 6128.47 | 6207.48 |
|  | … + species | 5904.56 | 5986.94 |
|  | … + ecoregion | 5859.53 | 5946.70 |
| *Anopheles* | Study number + site number | 5931.84 | 5961.68 |
|  | … + study block | 5884.59 | 6381.32 |
|  | … + study sample | 5828.39 | 6285.44 |
|  | … + species | 5475.25 | 6356.43 |
|  | … + ecoregion | 5477.65 | 6365.52 |
