# Supplementary material for: Differing taxonomic responses of mosquito vectors to anthropogenic land-use change in Latin America and the Caribbean
Source: PLoS Negl Trop Dis. 2023 Jul 14;17(7):e0011450. doi: 10.1371/journal.pntd.0011450 (PMC10348580; doi:10.1371/journal.pntd.0011450)
Supplement: S9 Table — Summary of components of total, Aedes and Anopheles abundance and species richness models in response to recent deforestation. The number of sites and site-level records in each model is shown. Only records at primary and secondary vegetation sites were included. (DOCX) [file pntd.0011450.s010.docx]

| **Model** | **Response** | **Random effects** | **Number of sites** | **Number of site-level records** |
| --- | --- | --- | --- | --- |
| *Aedes* abundance | log(adjusted abundance)+1 | study, site, species, study sample, ecoregion | 81 | 572 |
| *Anopheles* abundance | log(adjusted abundance)+1 | study, site, species, study sample, ecoregion | 167 | 526 |
| *Aedes* species richness | *Aedes* species richness | study, site, study sample, ecoregion | 36 | 50 |
| *Anopheles* species richness | *Anopheles* species richness | study, site, study sample, ecoregion | 89 | 101 |
