# Supplementary material for: Differing taxonomic responses of mosquito vectors to anthropogenic land-use change in Latin America and the Caribbean
Source: PLoS Negl Trop Dis. 2023 Jul 14;17(7):e0011450. doi: 10.1371/journal.pntd.0011450 (PMC10348580; doi:10.1371/journal.pntd.0011450)
Supplement: S10 Table — Number of sites included in the dataset by country and land-use type. (DOCX) [file pntd.0011450.s011.docx]

| **Country** | **Land-use type** | **Number of sites** |
| --- | --- | --- |
| Argentina | primary vegetation-minimal | 2 |
|  | secondary vegetation | 1 |
|  | managed | 2 |
|  | urban | 2 |
| Bolivia | primary vegetation-minimal | 1 |
|  | primary vegetation-substantial | 1 |
| Brazil | primary vegetation-minimal | 63 |
|  | primary vegetation-substantial | 21 |
|  | secondary vegetation | 19 |
|  | managed | 46 |
|  | urban | 11 |
| Colombia | primary vegetation-substantial | 3 |
|  | secondary vegetation | 1 |
|  | managed | 3 |
|  | urban | 1 |
| Ecuador | primary vegetation-minimal | 1 |
|  | urban | 1 |
| French Guiana | primary vegetation-minimal | 6 |
|  | primary vegetation-substantial | 4 |
|  | secondary vegetation | 4 |
|  | managed | 2 |
|  | urban | 2 |
| Mexico | primary vegetation-minimal | 4 |
|  | secondary vegetation | 3 |
|  | managed | 4 |
| Peru | primary vegetation-minimal | 41 |
|  | primary vegetation-substantial | 4 |
|  | secondary vegetation | 1 |
|  | managed | 20 |
|  | urban | 6 |
| Puerto Rico | primary vegetation-substantial | 1 |
| Saint Kitts and Nevis | primary vegetation-substantial | 1 |
|  | managed | 1 |
| Venezuela | primary vegetation-substantial | 1 |
|  | secondary vegetation | 3 |
|  | urban | 4 |
