# Supplementary material for: Differing taxonomic responses of mosquito vectors to anthropogenic land-use change in Latin America and the Caribbean
Source: PLoS Negl Trop Dis. 2023 Jul 14;17(7):e0011450. doi: 10.1371/journal.pntd.0011450 (PMC10348580; doi:10.1371/journal.pntd.0011450)
Supplement: S11 Table — List of Aedes and Anopheles mosquitoes (n = 91) included in models and number of abundance records per species. (DOCX) [file pntd.0011450.s012.docx]

| **Genus** | **Species** | **Number of records** |
| --- | --- | --- |
| Aedes | aegypti | 1859 |
| Aedes | albifasciatus | 50 |
| Aedes | albopictus | 1722 |
| Aedes | angustivittatus | 15 |
| Aedes | argyrothorax | 90 |
| Aedes | busckii | 20 |
| Aedes | crinifer | 62 |
| Aedes | dupreei | 5 |
| Aedes | epactius | 6 |
| Aedes | fluviatilis | 115 |
| Aedes | fulvithorax | 23 |
| Aedes | fulvus | 137 |
| Aedes | guerrero | 2 |
| Aedes | hastatus | 35 |
| Aedes | hortator | 27 |
| Aedes | infirmatus | 9 |
| Aedes | mediovitattus | 25 |
| Aedes | mediovittatus | 50 |
| Aedes | nubilus | 41 |
| Aedes | oligopistus | 34 |
| Aedes | patersoni | 8 |
| Aedes | pennai | 2 |
| Aedes | phaenonotus | 4 |
| Aedes | podographicus | 5 |
| Aedes | quadrivittatus | 32 |
| Aedes | rhyacophilus | 21 |
| Aedes | scapularis | 602 |
| Aedes | serratus | 548 |
| Aedes | taeniorhynchus | 159 |
| Aedes | terrens | 133 |
| Aedes | tormentor | 18 |
| Aedes | tortilis | 145 |
| Aedes | trivittatus | 4 |
| Anopheles | albimanus | 84 |
| Anopheles | albitarsis | 263 |
| Anopheles | allopha | 4 |
| Anopheles | apicimacula | 7 |
| Anopheles | aquasalis | 27 |
| Anopheles | argyritarsis | 129 |
| Anopheles | arthuri | 9 |
| Anopheles | atropos | 25 |
| Anopheles | bellator | 43 |
| Anopheles | benarrochi | 47 |
| Anopheles | bradleyi | 15 |
| Anopheles | braziliensis | 78 |
| Anopheles | calderoni | 4 |
| Anopheles | costai | 17 |
| Anopheles | cruzii | 132 |
| Anopheles | darlingi | 698 |
| Anopheles | deaneorum | 25 |
| Anopheles | dunhami | 44 |
| Anopheles | eiseni | 47 |
| Anopheles | evansae | 161 |
| Anopheles | fluminensis | 68 |
| Anopheles | forattinii | 17 |
| Anopheles | galvaoi | 85 |
| Anopheles | gilesi | 1 |
| Anopheles | goeldii | 41 |
| Anopheles | grabhamii | 25 |
| Anopheles | ininii | 12 |
| Anopheles | intermedius | 96 |
| Anopheles | janconnae | 21 |
| Anopheles | kompi | 15 |
| Anopheles | konderi | 9 |
| Anopheles | lanei | 20 |
| Anopheles | lutzii | 59 |
| Anopheles | maculipes | 9 |
| Anopheles | malefactor | 9 |
| Anopheles | marajoara | 51 |
| Anopheles | mattogrossensis | 226 |
| Anopheles | mediopunctatus | 136 |
| Anopheles | minor | 16 |
| Anopheles | neivai | 4 |
| Anopheles | neomaculipalpus | 9 |
| Anopheles | nimbus | 176 |
| Anopheles | nuneztovari | 171 |
| Anopheles | oryzalimnetes | 22 |
| Anopheles | oswaldoi | 183 |
| Anopheles | parvus | 25 |
| Anopheles | peryassui | 34 |
| Anopheles | pseudopunctipennis | 34 |
| Anopheles | pseudotibiamaculatus | 4 |
| Anopheles | punctimacula | 15 |
| Anopheles | rangeli | 86 |
| Anopheles | rondoni | 25 |
| Anopheles | shannoni | 27 |
| Anopheles | squamifemur | 9 |
| Anopheles | strodei | 187 |
| Anopheles | tibiamaculatus | 3 |
| Anopheles | triannulatus | 407 |
| Anopheles | vestitipennis | 40 |
