# Supplementary material for: Differing taxonomic responses of mosquito vectors to anthropogenic land-use change in Latin America and the Caribbean
Source: PLoS Negl Trop Dis. 2023 Jul 14;17(7):e0011450. doi: 10.1371/journal.pntd.0011450 (PMC10348580; doi:10.1371/journal.pntd.0011450)
Supplement: S12 Table — Posterior mean estimates, lower (2.5%) and upper (97.5%) credible intervals (CI) for land-use types in species richness models of Aedes and Anopheles mosquitoes. (DOCX) [file pntd.0011450.s013.docx]

| **Model** | **Land-use type** | **Mean** | **LCI** | **UCI** |
| --- | --- | --- | --- | --- |
| Total species richness | Primary vegetation – minimal (intercept) | 1.36 | 1.19 | 1.53 |
|  | Primary vegetation - substantial | -0.14 | -0.32 | 0.04 |
|  | Secondary vegetation - combined | -0.01 | -0.18 | 0.16 |
|  | Managed - combined | 0.04 | -0.08 | 0.17 |
|  | Urban - combined | -0.48 | -0.65 | -0.31 |
| *Aedes* species richness | Primary vegetation – minimal (intercept) | 0.95 | 0.74 | 1.16 |
|  | Primary vegetation - substantial | -0.12 | -0.39 | 0.15 |
|  | Secondary vegetation - combined | 0.01 | -0.24 | 0.27 |
|  | Managed - combined | -0.11 | -0.39 | 0.16 |
|  | Urban - combined | -0.31 | -0.56 | -0.05 |
| *Anopheles* species richness | Primary vegetation – minimal (intercept) | 1.12 | 0.92 | 1.31 |
|  | Primary vegetation - substantial | -0.13 | -0.37 | 0.10 |
|  | Secondary vegetation - combined | -0.03 | -0.26 | 0.20 |
|  | Managed - combined | 0.06 | -0.09 | 0.21 |
|  | Urban - combined | -0.43 | -0.69 | -0.15 |
