# Supplementary material for: Differing taxonomic responses of mosquito vectors to anthropogenic land-use change in Latin America and the Caribbean
Source: PLoS Negl Trop Dis. 2023 Jul 14;17(7):e0011450. doi: 10.1371/journal.pntd.0011450 (PMC10348580; doi:10.1371/journal.pntd.0011450)
Supplement: S13 Table — Posterior mean estimates, lower (2.5%) and upper (97.5%) credible intervals (CI) for land-use types in abundance models of Aedes and Anopheles mosquitoes. (DOCX) [file pntd.0011450.s014.docx]

| **Model** | **Land-use type** | **Mean** | **LCI** | **UCI** |
| --- | --- | --- | --- | --- |
| Total abundance | Primary vegetation – minimal (intercept) | 0.25 | -0.72 | 1.11 |
|  | Primary vegetation - substantial | -0.04 | -0.19 | 0.11 |
|  | Secondary vegetation - combined | 0.02 | -0.15 | 0.18 |
|  | Managed - combined | 0.08 | -0.04 | 0.21 |
|  | Urban - combined | -0.09 | -0.19 | 0.01 |
| *Aedes* abundance | Primary vegetation – minimal (intercept) | 0.51 | -0.58 | 1.50 |
|  | Primary vegetation - substantial | -0.01 | -0.20 | 0.18 |
|  | Secondary vegetation - combined | 0.04 | -0.16 | 0.24 |
|  | Managed - combined | -0.06 | -0.28 | 0.16 |
|  | Urban - combined | -0.06 | -0.17 | 0.04 |
| *Anopheles* abundance | Primary vegetation – minimal (intercept) | 0.59 | 0.33 | 0.84 |
|  | Primary vegetation - substantial | -0.12 | -0.28 | 0.04 |
|  | Secondary vegetation - combined | -0.05 | -0.21 | 0.11 |
|  | Managed - combined | 0.11 | 0.00 | 0.22 |
|  | Urban - combined | -0.14 | -0.25 | -0.02 |
