# Supplementary material for: Differing taxonomic responses of mosquito vectors to anthropogenic land-use change in Latin America and the Caribbean
Source: PLoS Negl Trop Dis. 2023 Jul 14;17(7):e0011450. doi: 10.1371/journal.pntd.0011450 (PMC10348580; doi:10.1371/journal.pntd.0011450)
Supplement: S14 Table — Posterior mean estimates, lower (2.5%) and upper (97.5%) credible intervals (CI) for land-use types in abundance models of four Aedes and four Anopheles mosquitoes. (DOCX) [file pntd.0011450.s015.docx]

| **Model** | **Land-use type** | **Mean** | **LCI** | **UCI** |
| --- | --- | --- | --- | --- |
| *Aedes aegypti* | Primary vegetation – minimal (intercept) | 1.65 | 0.91 | 2.39 |
|  | Primary vegetation - substantial | -0.54 | -1.06 | -0.03 |
|  | Secondary vegetation - combined | -0.82 | -1.39 | -0.25 |
|  | Managed - combined | 1.08 | 0.46 | 1.70 |
|  | Urban - combined | 0.18 | -0.07 | 0.42 |
| *Aedes albopictus* | Primary vegetation – minimal (intercept) | 0.87 | 0.16 | 1.59 |
|  | Primary vegetation - substantial | 0.67 | 0.23 | 1.09 |
|  | Secondary vegetation - combined | 0.52 | 0.05 | 0.98 |
|  | Managed - combined | 0.40 | -0.48 | 1.28 |
|  | Urban - combined | 0.10 | -0.11 | 0.31 |
| *Aedes scapularis* | Primary vegetation – minimal (intercept) | 1.83 | 1.04 | 2.63 |
|  | Primary vegetation - substantial | -0.59 | -0.85 | -0.32 |
|  | Secondary vegetation - combined | 0.08 | -0.18 | 0.35 |
|  | Managed - combined | -0.07 | -0.35 | 0.21 |
|  | Urban - combined | -0.02 | -0.13 | 0.09 |
| *Aedes serratus* | Primary vegetation – minimal (intercept) | 1.86 | 1.02 | 2.70 |
|  | Primary vegetation - substantial | -0.93 | -1.41 | -0.46 |
|  | Secondary vegetation - combined | 0.24 | -0.25 | 0.73 |
|  | Managed - combined | -1.08 | -1.58 | -0.59 |
|  | Urban - combined | -0.11 | -0.31 | 0.08 |
| *Anopheles albimanus* | Primary vegetation – minimal (intercept) | 0.41 | -0.92 | 1.72 |
|  | Primary vegetation - substantial | 0.86 | -0.63 | 2.35 |
|  | Secondary vegetation - combined | 0.02 | -1.54 | 1.58 |
|  | Managed - combined | 0.47 | -1.09 | 2.02 |
|  | Urban - combined | 0.46 | -1.23 | 2.20 |
| *Anopheles albitarsis* | Primary vegetation – minimal (intercept) | 0.80 | -0.01 | 1.62 |
|  | Primary vegetation - substantial | -0.05 | -0.76 | 0.68 |
|  | Secondary vegetation - combined | 0.38 | -0.52 | 1.27 |
|  | Managed - combined | 0.97 | 0.30 | 1.65 |
|  | Urban - combined | -0.02 | -0.92 | 0.88 |
| *Anopheles darlingi* | Primary vegetation – minimal (intercept) | 1.40 | 0.81 | 1.98 |
|  | Primary vegetation - substantial | -0.21 | -0.79 | 0.36 |
|  | Secondary vegetation - combined | 0.37 | -0.33 | 1.06 |
|  | Managed - combined | 0.21 | -0.12 | 0.54 |
|  | Urban - combined | -0.13 | -0.80 | 0.54 |
| *Anopheles nuneztovari* | Primary vegetation – minimal (intercept) | 0.81 | 0.30 | 1.32 |
|  | Primary vegetation - substantial | -0.19 | -0.80 | 0.42 |
|  | Secondary vegetation - combined | 0.22 | -0.48 | 0.90 |
|  | Managed - combined | -0.13 | -0.71 | 0.45 |
|  | Urban - combined | -0.42 | -1.06 | 0.21 |
