# Supplementary material for: Differing taxonomic responses of mosquito vectors to anthropogenic land-use change in Latin America and the Caribbean
Source: PLoS Negl Trop Dis. 2023 Jul 14;17(7):e0011450. doi: 10.1371/journal.pntd.0011450 (PMC10348580; doi:10.1371/journal.pntd.0011450)
Supplement: S15 Table — Number of unique sites by land-use type where Aedes and Anopheles mosquito species were recorded. (DOCX) [file pntd.0011450.s016.docx]

| **Species name** | **Land-use type** | **Number of sites** |
| --- | --- | --- |
| *Aedes aegypti* | managed | 10 |
|  | primary vegetation | 76 |
|  | secondary vegetation | 22 |
|  | urban | 125 |
| *Aedes albopictus* | managed | 6 |
|  | primary vegetation | 95 |
|  | secondary vegetation | 29 |
|  | urban | 128 |
| *Aedes scapularis* | managed | 19 |
|  | primary vegetation | 97 |
|  | secondary vegetation | 24 |
|  | urban | 90 |
| *Aedes serratus* | managed | 14 |
|  | primary vegetation | 86 |
|  | secondary vegetation | 18 |
|  | urban | 88 |
| *Anopheles albimanus* | managed | 4 |
|  | primary vegetation | 10 |
|  | secondary vegetation | 5 |
|  | urban | 3 |
| *Anopheles albitarsis* | managed | 17 |
|  | primary vegetation | 35 |
|  | secondary vegetation | 8 |
|  | urban | 8 |
| *Anopheles darlingi* | managed | 37 |
|  | primary vegetation | 105 |
|  | secondary vegetation | 10 |
|  | urban | 18 |
| *Anopheles nuneztovari* | managed | 11 |
|  | primary vegetation | 26 |
|  | secondary vegetation | 9 |
|  | urban | 9 |
