# Supplementary figures and images for: Differing taxonomic responses of mosquito vectors to anthropogenic land-use change in Latin America and the Caribbean
Source: PLoS Negl Trop Dis. 2023 Jul 14;17(7):e0011450. doi: 10.1371/journal.pntd.0011450 (PMC10348580; doi:10.1371/journal.pntd.0011450)

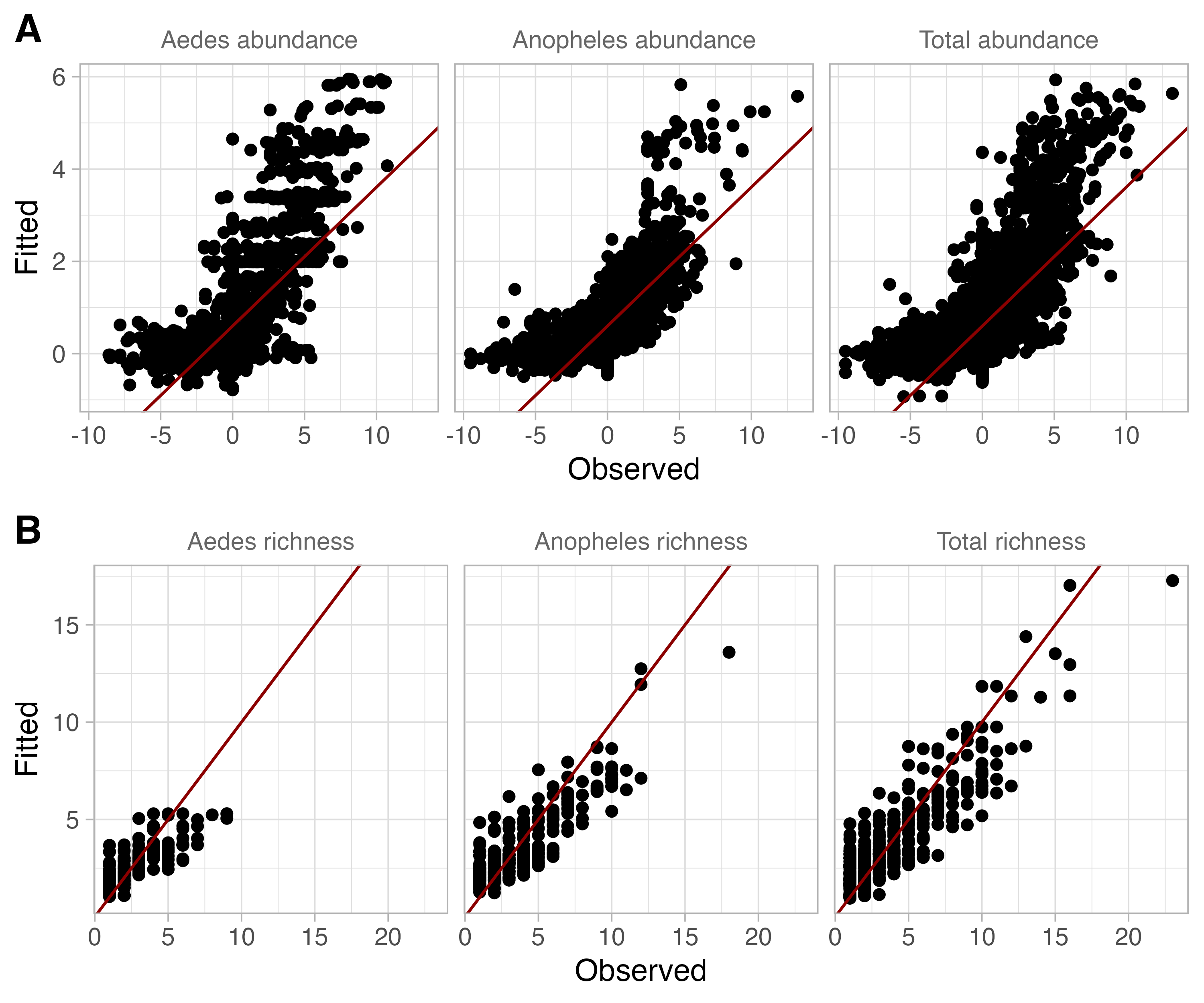

Supplement: S2 Fig — Observed and fitted model A) abundance (log +1) and B) species richness in models of total and Aedes and Anopheles mosquitoes. Red line represents the expectation if observed values equal fitted values. (TIF) [file pntd.0011450.s018.tif]

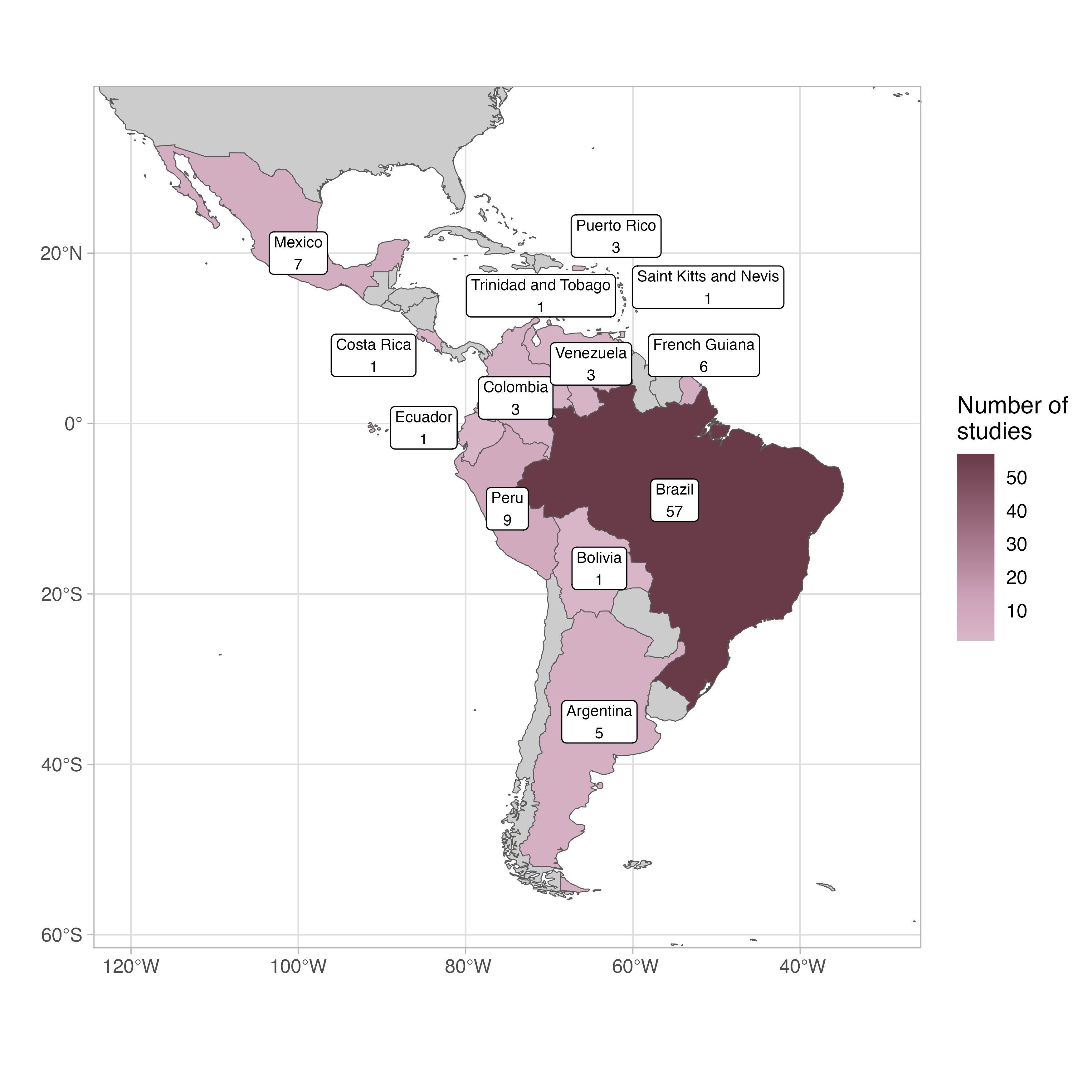

Supplement: S3 Fig — Number of included studies by country in Latin America and the Caribbean. The total number of included studies was 93. Base map sourced from rnaturalearth (50). (TIF) [file pntd.0011450.s019.tif]

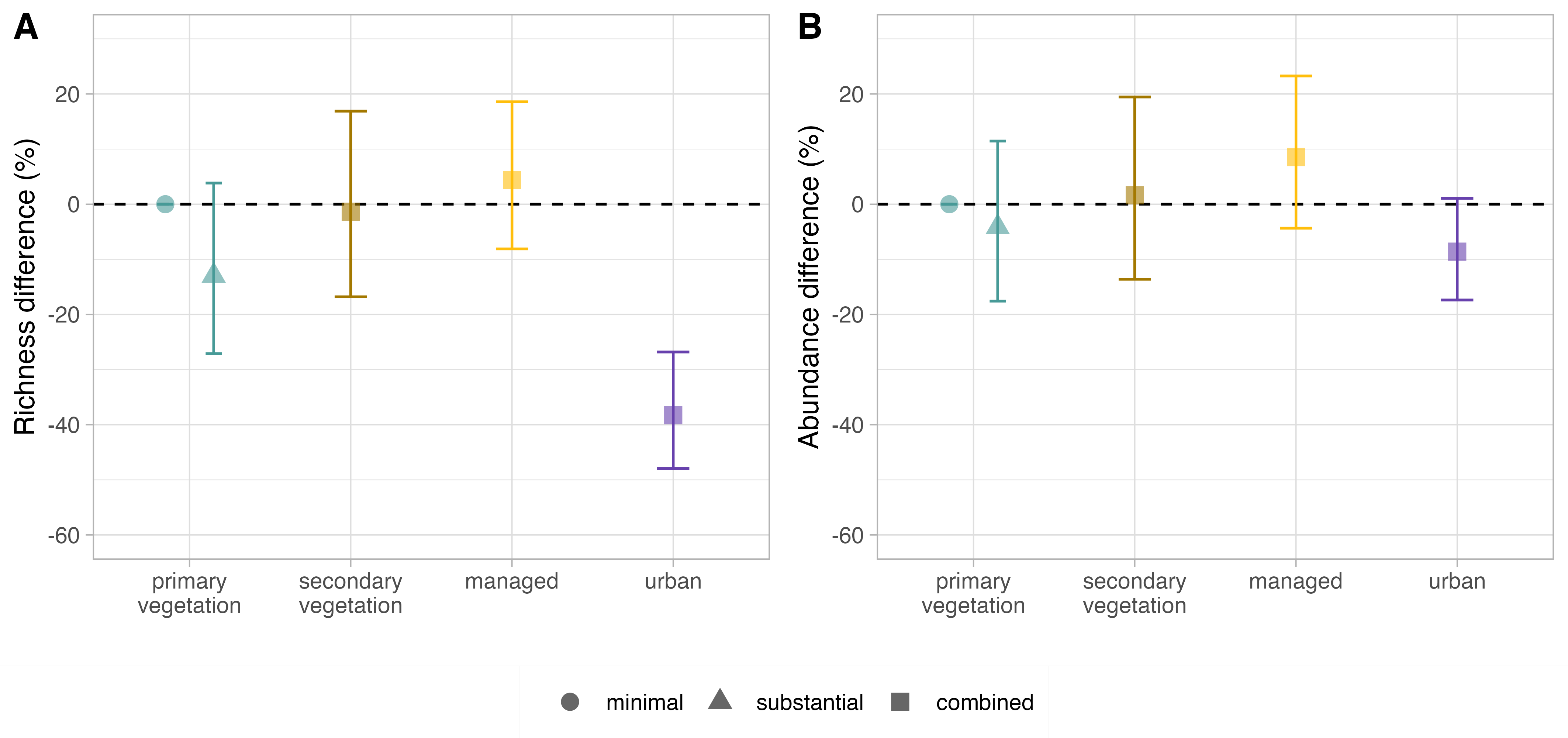

Supplement: S4 Fig — Total (Aedes and Anopheles) mosquito richness (A) and abundance (B) responses to land-use types with minimal (circles), substantial (triangles) and combined (squares) use intensities. Effect sizes were adjusted to a percentage by expressing each mean fixed effect and 95% credible intervals as a percentage of the baseline (primary vegetation minimal use, shown as zero). Intensity levels for secondary vegetation, managed and urban land uses were aggregated due to a lack of data representation. (TIF) [file pntd.0011450.s020.tif]

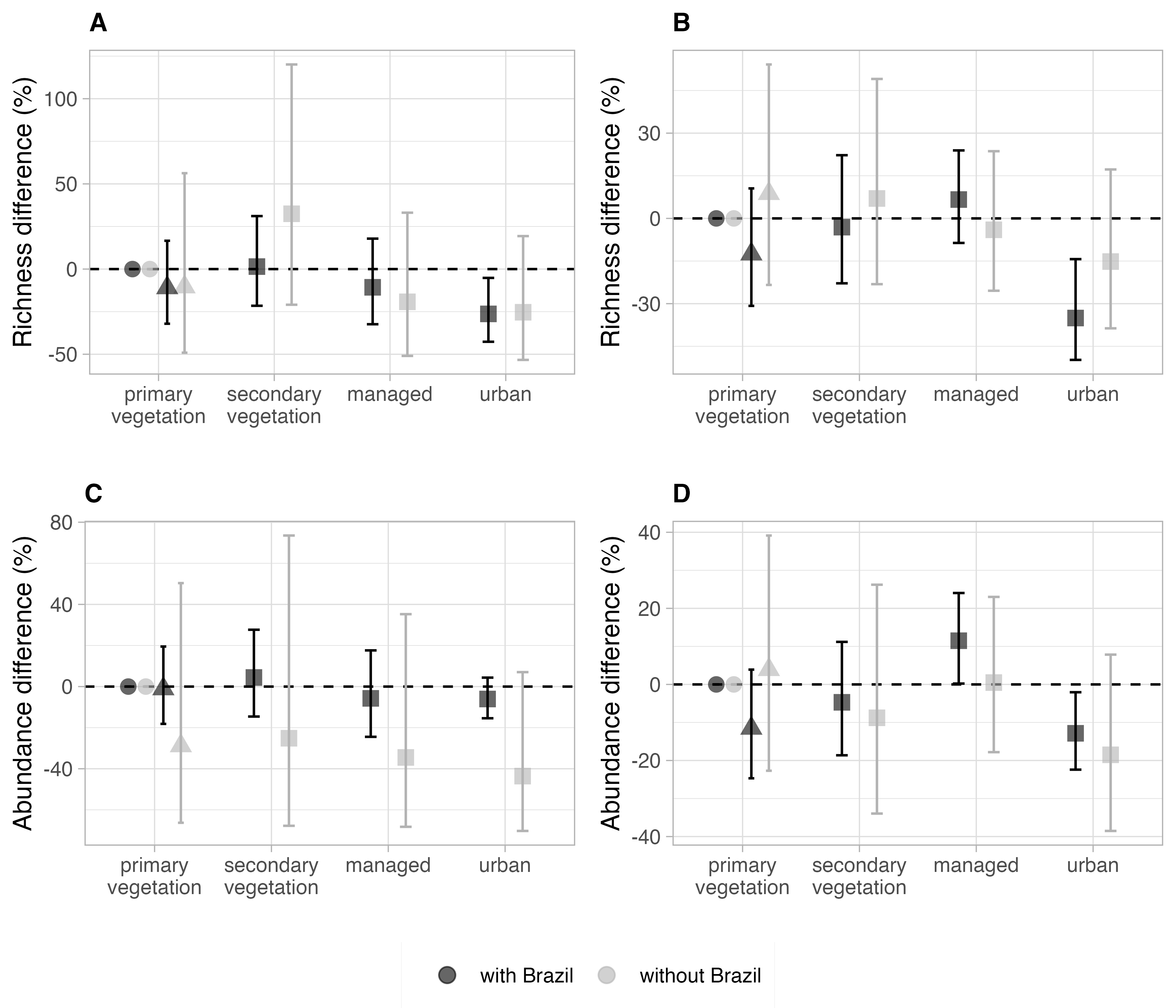

Supplement: S5 Fig — Response of Aedes (A, C) and Anopheles (B, D) mosquitoes to land-use type and intensity excluding sites from Brazil. Dark grey estimates show the genus-level richness (A-B) and abundance (C-D) models with all the data and the light grey estimates show modelled estimates excluding sites from Brazil. Effect sizes were adjusted to a percentage by expressing each mean fixed effect and 95% credible intervals as a percentage of the baseline (primary vegetation minimal use, shown as zero). Intensity levels for secondary vegetation, managed and urban land uses were aggregated due to a lack of data representation. (TIF) [file pntd.0011450.s021.tif]

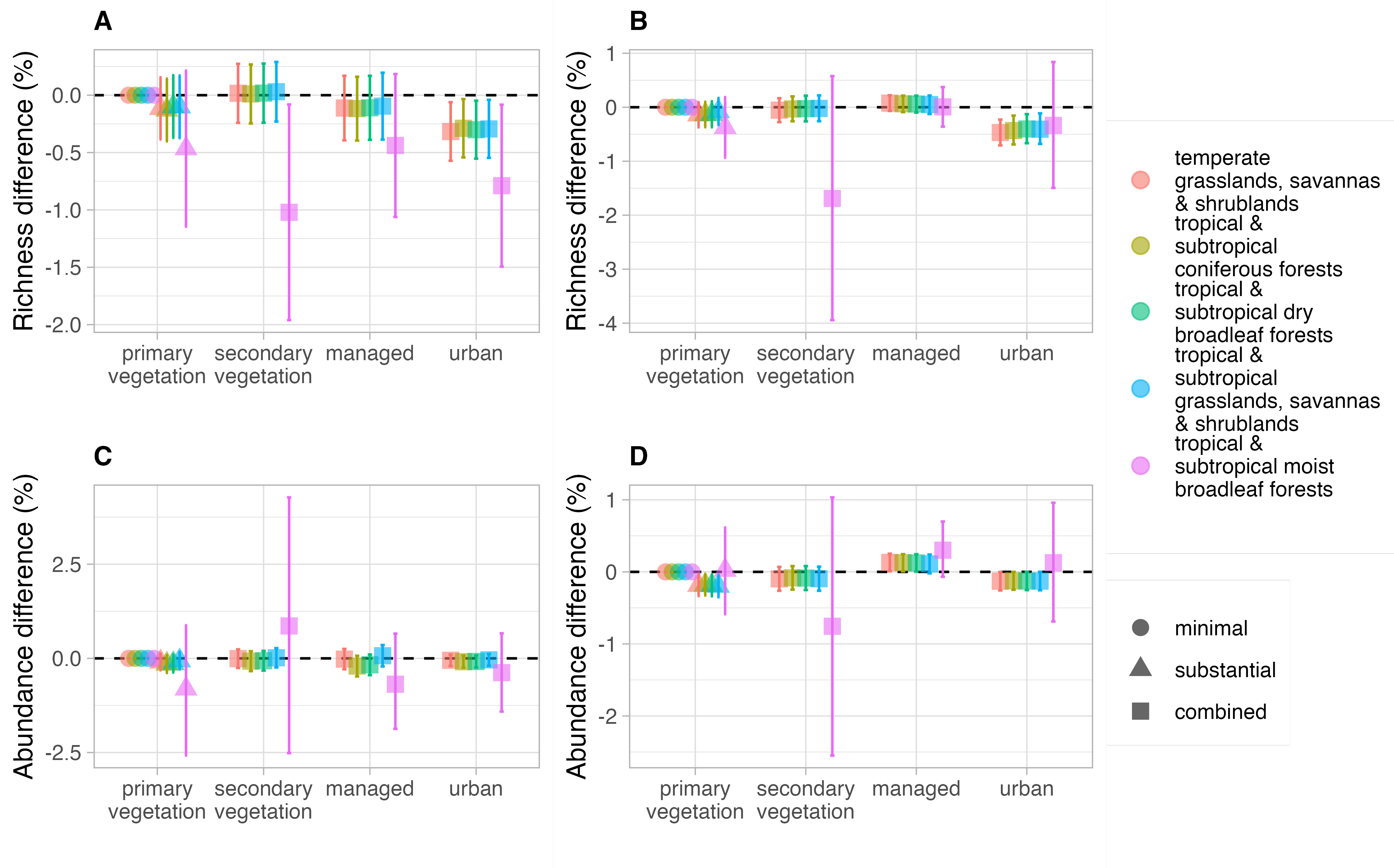

Supplement: S6 Fig — Response of Aedes (A, C) and Anopheles (B, D) mosquito species richness (A-B) and abundance (C-D) to land-use type and intensity excluding each ecoregion in turn. Colours represent each ecoregion that was excluded. Effect sizes were adjusted to a percentage by expressing each mean fixed effect and 95% credible intervals as a percentage of the baseline (primary vegetation minimal use, shown as zero). Intensity levels for secondary vegetation, managed and urban land uses were aggregated due to a lack of data representation. Both abundance and species richness were highly sensitive to rainforest sites (pink—tropical and subtropical moist broadleaf forests). (TIF) [file pntd.0011450.s022.tif]

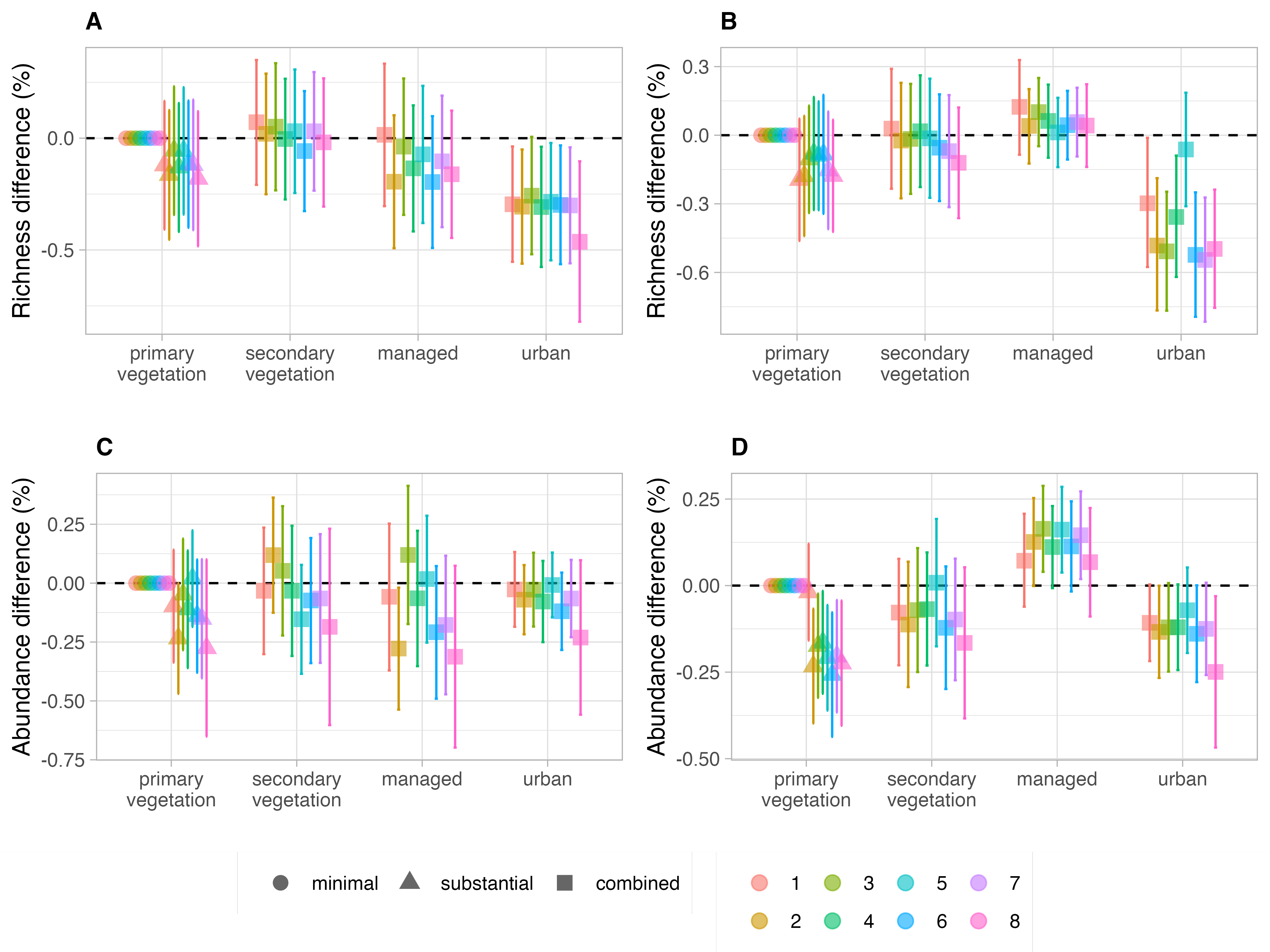

Supplement: S7 Fig — Response of Aedes (A, C) and Anopheles (B, D) mosquito species richness (A-B) and abundance (C-D) to land-use type and intensity excluding 12.5% of the data at time. Colours represent each data group. Effect sizes were adjusted to a percentage by expressing each mean fixed effect and 95% credible intervals as a percentage of the baseline (primary vegetation minimal use, shown as zero). Intensity levels for secondary vegetation, managed and urban land uses were aggregated due to a lack of data representation. (TIF) [file pntd.0011450.s023.tif]

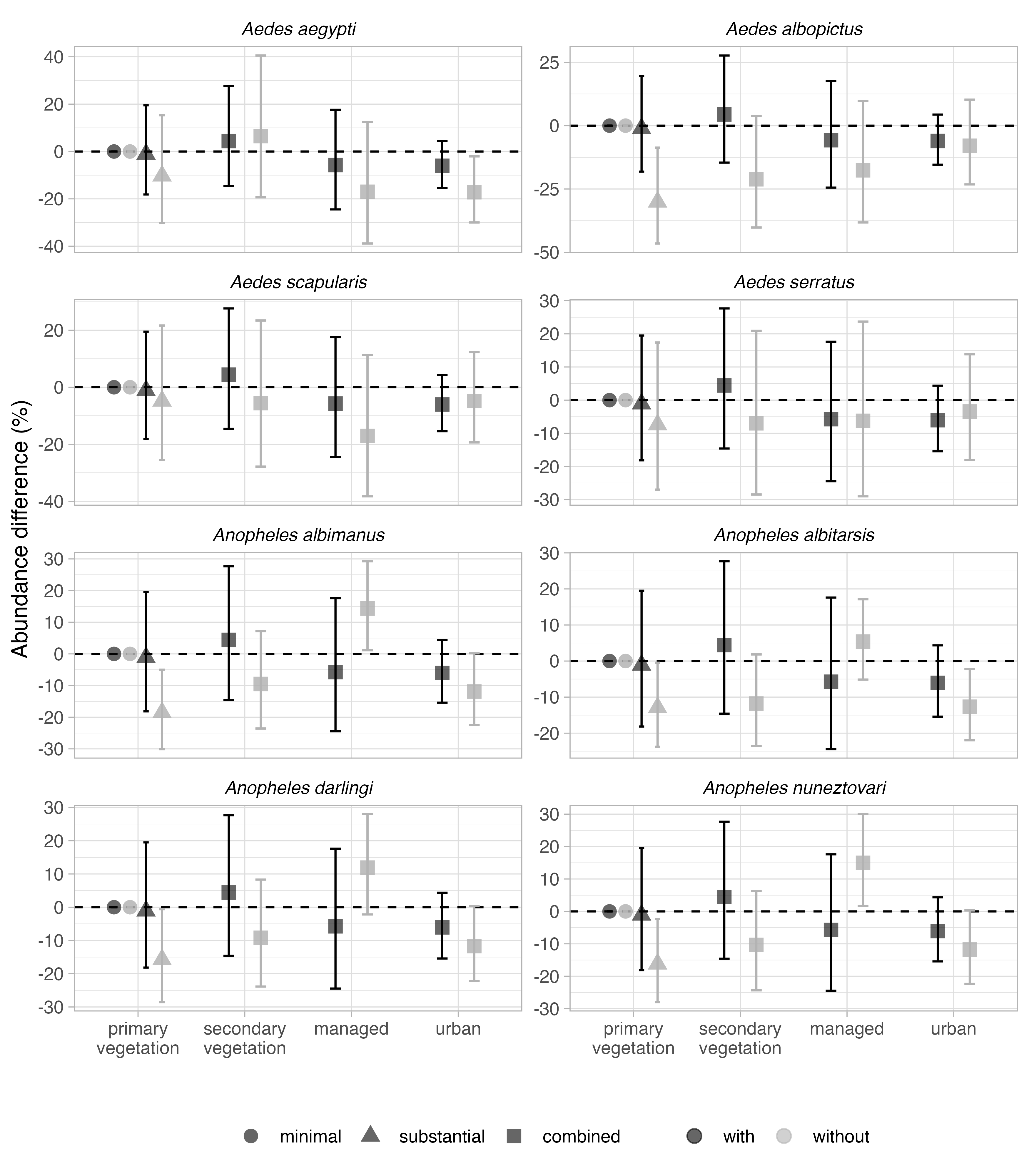

Supplement: S8 Fig — Response of Aedes (A) and Anopheles (B) mosquito abundance to land-use type and intensity excluding influential species. Dark grey estimates show the genus-level abundance model with all the data and the light grey estimates show modelled estimates excluding data for each species. For each genus, the four most represented species in the dataset were selected. Effect sizes were adjusted to a percentage by expressing each mean fixed effect and 95% credible intervals as a percentage of the baseline (primary vegetation minimal use, shown as zero). Intensity levels for secondary vegetation, managed and urban land uses were aggregated due to a lack of data representation. (TIF) [file pntd.0011450.s024.tif]
