# Supplementary material for: Differing taxonomic responses of mosquito vectors to anthropogenic land-use change in Latin America and the Caribbean
Source: PLoS Negl Trop Dis. 2023 Jul 14;17(7):e0011450. doi: 10.1371/journal.pntd.0011450 (PMC10348580; doi:10.1371/journal.pntd.0011450)
Supplement: Attachment — Submitted filename: Review_AP.pdf [file pntd.0011450.s026.pdf]

General comments:

This study aims to characterize the effects of land-use changes on the abundance and diversity of mosquitoes. The approach is very interesting given the amount of data available. However, I find that the manuscript is very focused on one result: All mosquito species do not respond in the same way to land-use changes, while this result is already well known and synthesized in recent reviews:

BURKETT-CADENA, N.D. & VITTOR, A.Y. (2018) Deforestation and vector-borne disease: Forest conversion favors important mosquito vectors of human pathogens. *Basic and Applied Ecology* **26**, 101–110.

PERRIN, A., GLAIZOT, O. & CHRISTE, P. (2022) Worldwide impacts of landscape anthropization on mosquito abundance and diversity: A meta-analysis. *Global Change Biology* **28**, 6857–6871.

SALLAM, M., FIZER, C., PILANT, A. & WHUNG, P.-Y. (2017) Systematic review: Land cover, meteorological, and socioeconomic determinants of *Aedes* mosquito habitat for risk mapping. *International Journal of Environmental Research and Public Health* **14**, 1230.

Conversely, a result that I found very interesting but not really highlighted and detailed in the manuscript is the effect of the dynamic of deforestation on the abundance of several species, such as *An. darlingi*. When deforestation is rapid/severe/high (in the 5 years before the sampling), species abundance increases more than when deforestation is slow/low. This is a surprising result because for the same species the response to land-use changes was not significant (no difference in abundance between urban and forest habitat). This therefore suggests that the dynamic of land-use change may be more important than the change itself in explaining the observed patterns. This conclusion should be further investigated and discussed in the manuscript because this is an important information for conservation biology and landscape management.

Specific comments:

L16: To avoid the repetition of 'including', I propose to replace 'including dengue and malaria' by '(e.g., dengue and malaria)'.

L30: I suggest to homogenize land use change/land-use change/land-use changes throughout the manuscript.

L150: Are there studies included in the analyzes whose sampling effort was not reported? If yes, how many?

L167: How is the use of multiple capture methods within a study modeled?

L169: ‘for each unique species at each site’ is not clear for me. In the supplementary materials, it is only a random effect ‘species’, in this case I suggest to remove ‘at each site’.

L175-183: Should the results be given with all the random effects included in the models? The majority of them are included to take into account the non-independence of the data, whether they have a significant effect or not.

L272: Repetition (L190-191).

L311-312: I suggest to give this information for all species since it could be interesting for the reader to see the balance or imbalance of sampling among species and among land-use types (either in an additional figure/table or directly integrated into the figures of the manuscript).

L318: ‘In contrast to spatial responses of mosquito biodiversity’. The following results do not contrast with the previous result, they are rather in agreement: the first shows a decline in

mosquito richness and abundance in urban areas, and the second shows a negative effect of deforestation.
L318-331: Is it really temporal? Here, several sites with different levels of deforestation in the last 5 years are compared. For a temporal approach, several records are needed within the same sampling site over time and linked to deforestation which acts at the same time. Rather than temporal deforestation, I would use deforestation intensity, severity, rapidity or something like that. In fact, the Figure 4 showed that the greater the deforestation in previous years following the sampling, the greater the abundance and richness of mosquitoes. Therefore, 'fast' deforestation does not have the same effect as 'slow' deforestation. This is an original and interesting result.
L360: This is the subject of several reviews published these last years (Sallam *et al.*, 2017; Burkett-Cadena & Vittor, 2018; Perrin, Glaizot & Christe, 2022)
L372-398: These three paragraphs deal with the importance of considering species-specific responses to land-use changes, but this result is already known in the literature. Could the results presented in this study allow us to go further? If so, I would rather discuss that, if not, I would synthesize these three paragraphs.
L403: More importantly, it is not the deforestation but the intensity of forest loss in the years before the sampling that seems to affect its abundance (the dynamic of deforestation is therefore highly important).
