# Supplementary material for: Differing taxonomic responses of mosquito vectors to anthropogenic land-use change in Latin America and the Caribbean
Source: PLoS Negl Trop Dis. 2023 Jul 14;17(7):e0011450. doi: 10.1371/journal.pntd.0011450 (PMC10348580; doi:10.1371/journal.pntd.0011450)
Supplement: Attachment — Submitted filename: PNTD-D-22-01333_DIO comments.pdf [file pntd.0011450.s027.pdf]

PLOS Neglected Tropical Diseases

Differing taxonomic responses of mosquito vectors to anthropogenic land-use change in Latin America and the Caribbean

--Manuscript Draft--

**Manuscript Number:**	PNTD-D-22-01333
**Full Title:**	Differing taxonomic responses of mosquito vectors to anthropogenic land-use change in Latin America and the Caribbean
**Short Title:**	Mosquito vector responses to anthropogenic land-use change
**Article Type:**	Research Article
**Keywords:**	dengue; malaria; mosquito; land use; biodiversity; vectors; environmental change; urbanisation
**Abstract:**	Anthropogenic land-use change, such as deforestation and urban development, can affect the emergence and re-emergence of mosquito-borne diseases, including dengue and malaria, by creating more favourable vector habitat. There has been a limited assessment of how mosquito vectors respond to land-use changes, including differential species responses. This information could help design optimal disease control strategies. We compiled an extensive dataset of 10,004 Aedes and Anopheles mosquito abundance records across multiple land-use types at 632 sites in Latin America and the Caribbean. Using a Bayesian mixed effects modelling framework to account for between-study differences, we compared spatial differences in the abundance and species richness of mosquitoes across multiple land-use types, including agricultural and urban areas. We found inconsistent mosquito responses to anthropogenic land-use change, with pronounced genus- and species-specific responses. There were strong declines in Aedes (-26%) and Anopheles (-37%) species richness in urban areas, whilst abundance of some mosquito species, such as Aedes aegypti, was favoured by anthropogenic disturbance. When abundance records were coupled with remotely sensed forest loss data, we detected a strong positive response of dominant and secondary malaria vectors to recent deforestation. This highlights the importance of the temporal dynamics of land use change in driving disease risk and the value of large synthetic datasets for understanding changing disease risk with environmental change.
**Additional Information:**	
**Question**	**Response**
**Government Employee**<br><br>Are you or any of the contributing authors an employee of the United States government?<br><br>Manuscripts authored by one or more US Government employees are not copyrighted, but are licensed under a CC0 Public Domain Dedication, which allows unlimited distribution and reuse of the article for any lawful purpose. This is a legal requirement for US Government employees.<br><br>This will be typeset if the manuscript is	No - No authors are employees of the U.S. government.

accepted for publication.	
**Financial Disclosure**<br><br>Enter a financial disclosure statement that describes the sources of funding for the work included in this submission and the role the funder(s) played. This includes grants and any commercial funding of the work or authors.<br><br>This statement will be typeset if the manuscript is accepted for publication.<br><br>Review the submission guidelines and the instructions link below for detailed requirements and guidance.	IKF was supported by the Biological and Biotechnology Research Council (grant BB/M009513/1) and RL was supported by a Royal Society Dorothy Hodgkin fellowship. RG was supported by a Graduate Research Fellowship from University College London.
**Competing Interests**<br><br>On behalf of all authors, disclose any competing interests that could be perceived to bias this work.<br><br>This statement will be typeset if the manuscript is accepted for publication.<br><br>Review the instructions link below and PLOS NTDs' competing interests policy to determine what information must be disclosed at submission.	The authors declare no competing interest
**Data Availability**<br><br>Provide a **Data Availability Statement** in the box below. This statement should detail where the data used in this submission can be accessed. This statement will be typeset if the manuscript is accepted for publication.<br><br>Before publication, authors are required to make all data underlying their findings fully available, without restriction. Review our PLOS Data Policy page for detailed information on this policy. Instructions for	All data and code that support the findings of this study are available in the Github repository https://github.com/isabelfletcher/vectors_landuse

writing your Data Availability statement can be accessed via the Instructions link below.	

Powered by Editorial Manager® and ProduXion Manager® from Aries Systems Corporation

Manuscript including figures
Click here to access/download;Manuscript;vectors_landuse_IFletcher.docx

Differing taxonomic responses of mosquito vectors to anthropogenic land-use change in Latin America and the Caribbean

Isabel K. Fletcher[1,2]*, Rory Gibb[1,2,3], Rachel Lowe[1,2,4,5] & Kate E. Jones[3]

[1]Centre for Mathematical Modelling of Infectious Diseases, London School of Hygiene & Tropical Medicine, London, UK.

[2]Centre on Climate Change and Planetary Health, London School of Hygiene & Tropical Medicine, London, UK.

[3]Centre for Biodiversity and Environment Research, University College London, London, UK.

[4]Barcelona Supercomputing Center, Barcelona, Spain.

[5]Catalan Institution for Research and Advanced Studies, Barcelona, Spain.

*Correspondence to: Isabel Fletcher, Department of Infectious Disease Epidemiology, London School of Hygiene & Tropical Medicine, London, UK, Email: isabel.fletcher1@lshtm.ac.uk

Abstract

Anthropogenic land-use change, such as deforestation and urban development, can affect the emergence and re-emergence of mosquito-borne diseases, including dengue and malaria, by creating more favourable vector habitat. There has been a limited assessment of how mosquito vectors respond to land-use changes, including differential species responses. This information could help design optimal disease control strategies. We compiled an extensive dataset of 10,004 *Aedes* and *Anopheles* mosquito abundance records across multiple land-use types at 632 sites in Latin America and the Caribbean. Using a Bayesian mixed effects modelling framework to account for between-study differences, we compared spatial differences in the abundance and species richness of mosquitoes across multiple land-use types, including agricultural and urban areas. We found inconsistent mosquito responses to anthropogenic land-use change, with pronounced genus- and species-specific responses. There were strong declines in *Aedes* (-26%) and *Anopheles* (-37%) species richness in urban areas, whilst abundance of some mosquito species, such as *Aedes aegypti*, was favoured by anthropogenic disturbance. When abundance records were coupled with remotely sensed forest loss data, we detected a strong positive response of dominant and secondary malaria vectors to recent deforestation. This highlights the importance of the temporal dynamics of land use change in driving disease

risk and the value of large synthetic datasets for understanding changing disease risk with environmental change.

Keywords: malaria; dengue; mosquito; land use; biodiversity

Author Summary

An understanding of the response of disease vectors to anthropogenic activities can aid the control of mosquito-borne disease transmission. However, regional assessments of these responses are lacking, especially in areas where mosquito-borne diseases are emerging and re-emerging. We assembled a synthetic dataset of mosquito abundance and species richness across multiple land-use types in Latin America and the Caribbean, using data from peer-reviewed published studies. We then used this dataset to explore whether, compared to a baseline of minimal anthropogenic activity, mosquito abundance and species richness changed, depending on the type of land use. Overall, our results show a decline in mosquito biodiversity in urban areas. We detected distinct species-specific responses in abundance to land use, with some important disease vectors, such as *Aedes aegypti* increasing in abundance in anthropogenic environments. Finally, we also demonstrated by coupling our dataset with forest loss data that the abundance of dominant malaria vectors in region increases with deforestation.

Introduction

The global land system is facing mounting pressure from anthropogenic activities, including the conversion of natural environments for agricultural practices and urban development (1). Globally, 75% of land surface area has been transformed by anthropogenic activities, mostly through global net loss of forest cover and the expansion of global agricult[illegible] of ecosystems has devastating consequences for global biodiversity ~~(4)~~ and [illegible] influences the incidence and emergence of infectious diseases (~~5–8~~ 4 - 8). An improved understanding of how important disease vectors are impacted by land-use alterations is essential given current trends in land-use transformation and global heating ~~(9)~~, as well as the emergence and re-emergence of infectious diseases (~~10,11~~ 9-11).

I would recommend moving the in-text citation towards the end of the sentence.

Mosquito-borne diseases are particularly sensitive to ecological alterations resulting from land-use transformations, including changes in vector habitat availability and vector-human contact rates (12). For example, ecological changes caused by deforestation ~~for~~ due to agricultural development in the Brazilian Amazon, increase the abundance and biting of the principal

malaria vector, *Anopheles darlingi* (13,14). At intermediate levels of deforestation in agricultural frontier regions, greater amounts of forest edge habitat provide suitable conditions for the proliferation of *An. darlingi* mosquitoes. Subsequently, this elevates malaria risk in the early stages of land-use alterations (15–17). In addition to facilitating increases in habitat suitability for mosquito vectors, land-use change, such as agricultural development also increases human exposure to pathogen-carrying mosquitoes (18). These local-scale studies have demonstrated how land-use change can alter disease risk through modification of vector habitats. However, mosquito responses at a regional scale have not been previously identified. An assessment of how important vectors respond to land-use change and ecological alterations, such as deforestation, will be useful for designing control strategies that can be implemented at scale.

Given the distinct life-history characteristics and diversity of mosquito species (over 3,600 recognised Culicidae species (19)), it is likely that species will respond differently to land-use change. Urbanisation negatively impacts terrestrial biodiversity (4) and allows for synanthropic mosquitoes, which live in or near human dwellings, to persist in novel environments (20). This is due to the diverse range of aquatic habitats for mosquito breeding that exist in urban environments, such as water-storage containers, discarded plastic and drains (21). Increased provisioning of vector habitats in addition to the availability of human hosts have enabled synanthropic mosquitoes, such as the dengue vector *Ae. aegypti* and malaria vector *An. stephensi* to flourish in urban environments (22–25). In contrast, mosquito biodiversity is higher in rural, forested landscapes (26,27), with some mosquitoes exhibiting a preference for preserved forested habitats (28,29). Despite this understanding of species' habitat preferences, there is a limited understanding of whether mosquito species respond differentially to anthropogenic land-use change. In addition, several local-level studies have provided an increased mechanistic understanding of how habitat alterations such as deforestation favour important disease vectors (13). However, there has been limited assessment of whether consistent responses to deforestation can be detected regionally, and how the nature of these responses compares among mosquito species. Such assessments will be useful for developing effective mosquito control strategies that can be tailored to species behaviour, such as feeding and resting patterns.

In the Latin America and the Caribbean region (LAC) mosquito-borne diseases are a dynamic public health threat. Approximately five million dengue cases were reported in LAC in 2020

(30), 145 million people in the Americas are at risk of malaria and cases of yellow fever occur in 13 countries across the region, including Peru, Bolivia and Brazil (31). Globally, the Amazon rainforest is the largest reservoir of arboviruses (32) and is subject to intensifying human pressures, including the development of land for pasture and deforestation for soybean production (2,33). Alterations to natural landscapes have resulted in the rapid expansion of mosquito-borne diseases, such as dengue and yellow fever (34,35), and the rapid re-emergence of malaria in Venezuela (36).

Here, we develop a regional approach for assessing the response of *Aedes* and *Anopheles* mosquitoes to land use across LAC. We employ a systematic data search strategy to compile an extensive dataset of mosquito abundance records before using a comparative space-for-time approach to identify taxonomic responses to anthropogenic land-use change. We test to what extent taxonomic responses in mosquito abundance to spatial differences in anthropogenic land use differ. We also test whether mosquito species richness in human-dominated landscapes is reduced in comparison to areas not altered by human activity. Additionally, we investigate the temporal dynamics of land use change by testing for differences in mosquito species responses to recent deforestation, harmonising findings from local-level studies.

Methods

Mosquito biodiversity dataset construction and assignment of land-use categories

A dataset of *Aedes* and *Anopheles* mosquito species in LAC across multiple land-use types was built by extracting relevant abundance data from published studies using a systematic data search strategy (Supplementary Materials; Table S2). Dataset construction followed the methodology in Hudson *et al.* (2014) for the PREDICTS database (a global compilation of site-level ecological data across different land uses and land-use intensities). Species- and site-specific abundance data were extracted for each included study and information collated on the sampling methodology, the study area and site descriptions (Table S2). As with the PREDICTS database, each study site was nested to account for variation due to sampling methodology. Specifically, each record was assigned a study number (a unique paper), site number (a geographic location at which mosquito abundance was sampled), study block (a collection of sites within a distinct spatial cluster, to account for spatial autocorrelation within a study) and study sample (a sample with consistent sampling methodology, such as capture method and sample month) (Table S2).

Each sample site was assigned a land-use type (primary vegetation, secondary vegetation, managed or urban) and use intensity (minimal or substantial), adapted following criteria in Hudson *et al.* (2014) and Gibb *et al.* (2020) (Table S3). Sites were labelled according to the predominant land-use type as detailed in the site description within each study and use intensity was assigned based on how heavily sites were used. For example, sites sampled near or in buildings, or small rural villages, located in a biological reserve, research station or forested area, were labelled as primary vegetation with minimal or substantial use. Managed sites included those with plantations, pasture or cropland (8). For sites to be classified as urban, sites must have paved roads and significant impervious surface area. For analysis, land-use type and intensity were combined into a categorical variable. Minimal and substantial use intensities were retained for primary vegetation sites and due to a lack of data representation, use intensities for secondary vegetation, managed and urban sites were combined into a single category. This resulted in a categorical variable with five levels; primary vegetation-minimal, primary vegetation-substantial, secondary vegetation, managed and urban (Table S4).

Modelling the effects of land use on mosquito abundance and species richness

Bayesian mixed-effects models were formulated to assess the spatial variation mosquito biodiversity (species-level abundance and site-level species richness) across different land-use types (Table S5). Where a study reported sampling effort, which varied across sample sites within a study, raw species-level abundance measurements were divided by sampling effort to give an effort-corrected abundance measurement (4,38,39). Due to the high number of zero observations site-level species abundance measurements were overdispersed, so were log-transformed and subsequently modelled with a Gaussian likelihood. Site-level species richness (the number of uniquely named species sampled at each site) was modelled using a Poisson likelihood (4,8). Models were constructed for *Aedes* species abundance, *Anopheles* species and both species (all *Aedes* and *Anopheles* combined). Species-specific responses in abundance to land use were also examined by building separate models for four mosquito species per genus. The most represented (highest number of records) *Aedes* and *Anopheles* mosquito species in the dataset were selected (Table S6), whilst ensuring the selected species are implicated in transmission of at least one human disease (40,41). To ensure mosquito responses were not confounded by mosquito habitat preferences and ranges, the models for each species only included studies where the species was detected.

All models included a random intercept term for each study to account for variation between studies, including reporting methods and sampling methodologies (e.g., outdoor vs. indoor sampling and trapping method). A random effect for each study site was included to account for overdispersion due to site-level differences (37). Abundance models also included a random intercept for each unique species at each site, resulting in multiple observations per site. This allowed for species-specific variation in abundance that could result from differences in feeding and resting behaviour, meaning some species were easier to sample than others. Other random effects considered in the model structure included study block, used to account for spatial autocorrelation between sites within a study and study sample. Ecoregion, reflecting habitat types taken from WWF (2021) was also included as a random effect to account for the confounding effect of climate and habitat (37). The best-fitting random effects structure was selected by formulating iterative models of each response (abundance and richness) with the addition of each random effect (Table S7-8).

Model adequacy was then assessed using the Bayesian metrics, the deviance information criterion (DIC) (43) and the Watanabe-Akaike information criterion (WAIC) (44). Random effects were retained in the final model structure if the model was improved (assessed by a reduction in DIC and WAIC) with the addition of each random effect, although ecoregion was retained in all models to avoid the potential confounding influence of climate and habitat. Model fit was also assessed through visual inspection of fitted versus observed values (Figure S2). All models were implemented in a Bayesian framework, using R-INLA (45).

Species richness and abundance models were cross validated by testing the sensitivity of the fixed effects estimates to geographical and random subsampling. For geographical subsampling, models were fitted holding out data from Brazil where data coverage was highest. Models were also fitted to data excluding each ecoregion (n=6) at a time. Finally, for the random subsampling, eight models were fitted excluding 12.5% of the data at a time.

Modelling the impact of deforestation on mosquito biodiversity

To explore the temporal dimension of land use change, we compared species-specific mosquito responses to deforestation by combining abundance records with remotely-sensed deforestation data (46). Specifically, mosquito records from primary and secondary vegetation

sites in the dataset were used to explore differences in both *Aedes* and *Anopheles* species richness and abundance responses to deforestation. The Hansen dataset consists of spatially continuous annual estimates of forest loss derived from Landsat images, between 2000-2019 (46). For each unique primary and secondary vegetation site in the dataset, the percentage of forest loss within a 320 m buffer around each site was extracted. A 320 m buffer was used as an approximation of mosquito flight distance, which can range between 50 m and 50 km. Average *Aedes* and *Anopheles* flight distances range between 89 – 542 m (47) so the mean of these values was used. As the time since deforestation greatly influences mosquito dynamics and subsequent disease risk (16), the deforestation data was temporally matched with site-level mosquito abundance and richness records. An estimate of recent forest loss was obtained by using estimates from the last five years since the sampling start date at each site. Bayesian mixed-effects models for *Aedes* and *Anopheles* mosquito species richness and abundance were formulated including site-level proportional deforestation as a linear covariate. As with the land-use models, random effects for study number, site number, study sample and terrestrial ecoregion were also included (Table S9). Eight species-specific abundance models selected based on data representation were also formulated to test for individual responses to deforestation.

Results

Although Brazil is mentioned as one of the countries included in your article, other countries were not mentioned in the results. It'll be useful to name the countries and the number of studies so the reader get better idea of the proportion of studies per country and maybe type of site. My suggestion is to include a table in the results section with this information (ex. country, number of studies used, type of site/ ecosystem (urban, tropical rain forest, tropical dry forest, etc.), and author citation). It could serve as a quick reference for the reader. I still like the map and I think it still could be used.

Dataset of mosquito biodiversity and land use

The final mosquito abundance dataset comprised 10,004 records across 632 sites, from 93 studies identified in the systematic data search (Figure 1A). Most sampled sites were primary vegetation (46%, n=292; Figure 1A), which represented 37% of total records in the dataset (n=3,739). The dataset spanned 13 countries across the LAC region (Figure 1A; Figure S3) and coverage was highest in Brazil (69% of records, n=6,870), and in biodiversity hotspots such as the Amazon basin (68% of total sites, n=431; Figure 1B) and Atlantic Forest (18% of total sites, n=111; Figure 1A). The dataset spanned six terrestrial ecoregions (Figure S6), the majority of which were in the Amazon and were forested ecoregions (89% of total sites, n=564; Figure 1B). The dataset included 91 species (Table S10), of which 36% (n=33) were *Aedes* species and 64% (n=58) were *Anopheles* species (Figure 1C).

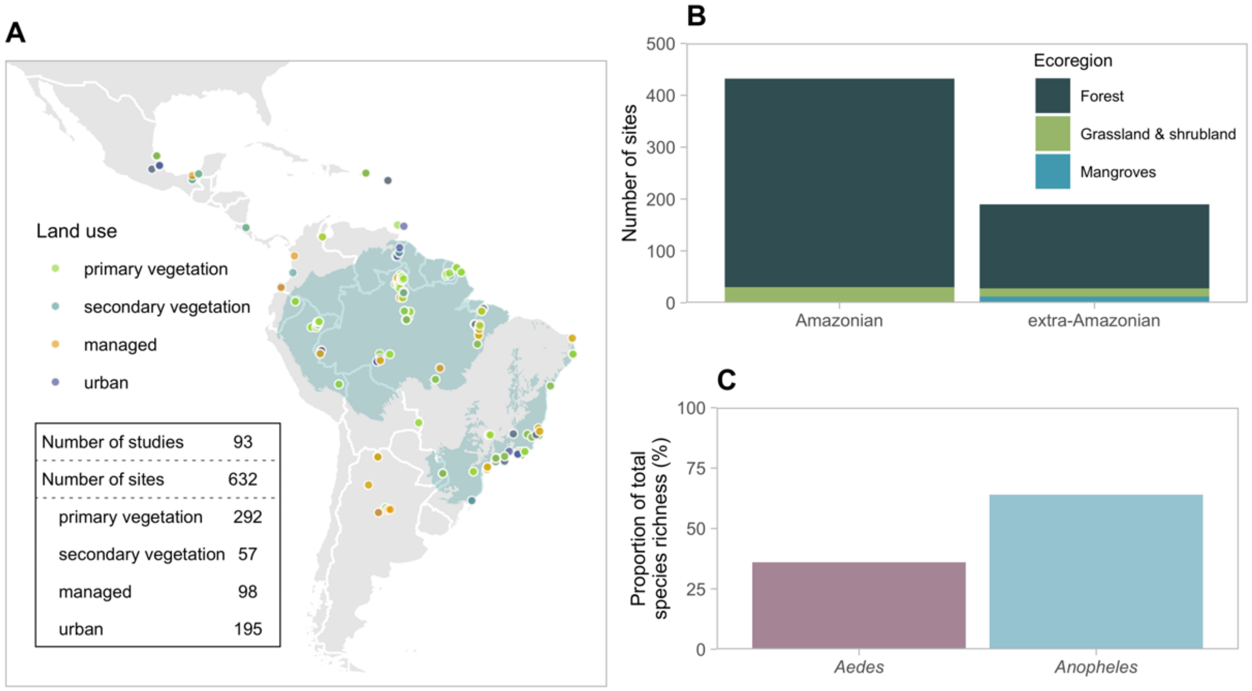

Figure 1. Dataset of *Aedes* and *Anopheles* mosquito biodiversity in Latin America and the Caribbean. Geographical location (points) of surveyed sites (n=632) and their predominant land-use type across 93 collated studies (A). Colours represent the four land-use types: primary vegetation (green), secondary vegetation (blue), managed (orange) and urban (purple). Green shading on the map shows the Amazon basin and Atlantic Forest. The number of surveyed sites across broadly defined terrestrial ecoregions (forests, grassland and shrubland, and mangroves) are shown for Amazonian and extra-Amazonian regions (the remaining LAC region) (B). Proportion (%) of unique species (species richness) across total species richness in the dataset (C).

Effect of land use on mosquito species richness and abundance

With the exception of urban areas, we found minimal evidence of consistent effects of land use on mosquito abundance and species richness diversity (Figure 2A; Figure S4A; Table S11). *Aedes* mosquito species richness was reduced by 26% in urban areas, compared to the primary vegetation minimal use baseline (95% CI: -42.7% to -5.2%; Figure 2A). *Anopheles* mosquito species richness demonstrated a larger 37% decline in richness (95% CI: -50.8% to -20.6%) and there was a trend towards increased *Anopheles* richness in managed areas, although the credible intervals crossed zero (Figure 2A). Relative to primary vegetation, *Anopheles* mosquitoes in urban sites experienced a significant 13% (95% CI: -22.4% to -2.1%) reduction in abundance and there was also a trend towards decreased *Aedes* abundance in urban sites (Figure 2B; Table S12). In contrast, abundance of *Anopheles* mosquitoes increased by 11% in managed sites (95% CI: 0.3% - 24.0%). There was a minimal effect of other land-use types on

overall *Aedes* and *Anopheles* abundance. Total *Aedes* and *Anopheles* mosquito species richness was 37% lower in urban landscapes (95% CI: -50.8% to -20.6%), and there was no significant effect of land-use type on total mosquito abundance (Figure S4B).

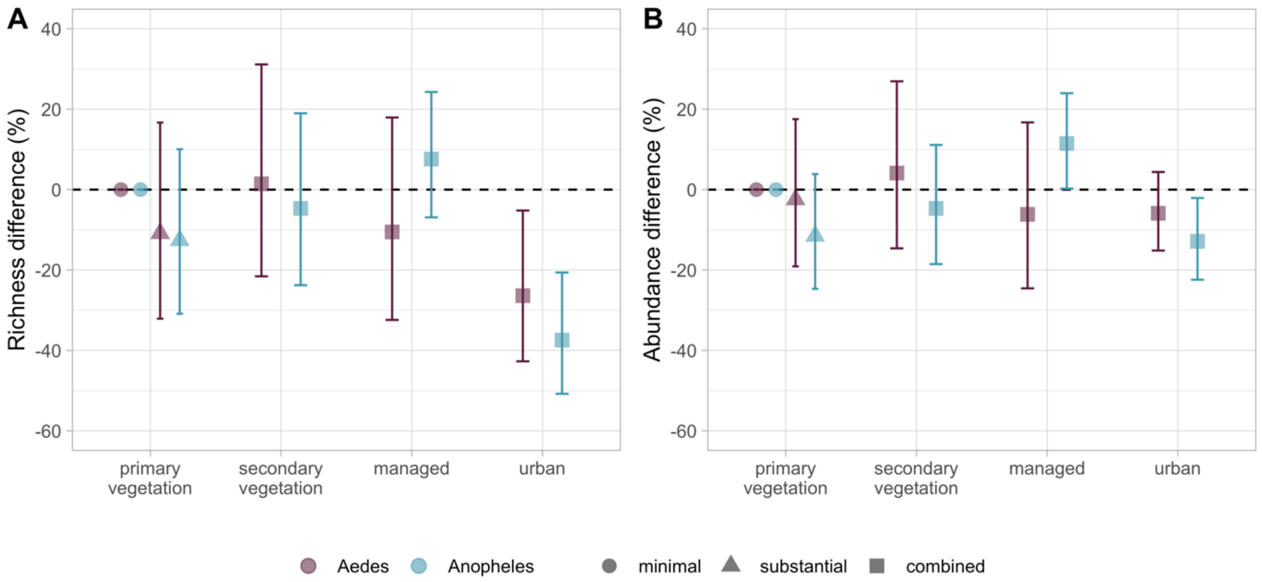

Figure 2. Responses of mosquito species richness and abundance to land-use type and intensity. *Aedes* (purple) and *Anopheles* (blue) mosquito species richness (A) and abundance (B) responses to land-use types with minimal (circles), substantial (triangles) and combined (squares) use intensities. Effect sizes were adjusted to a percentage by expressing each mean fixed effect and 95% credible intervals as a percentage of the baseline (primary vegetation minimal use, shown as zero). Intensity levels for secondary vegetation, managed and urban land uses were aggregated due to a lack of data representation.

Both species richness and abundance models were broadly robust to geographical subsampling, although there were higher levels of uncertainty in abundance and richness estimates when data from Brazil were excluded from the models (Figure S5). Urban estimates were particularly sensitive to holding out data from Brazil, likely because of the high number of Brazilian urban sites (Figure 1A). We also found that both the species richness models were highly influenced by sites from tropical rainforests, highlighting the need for more representative sampling outside this ecoregion (Figure S6). Finally, abundance and species richness responses were largely robust to random subsampling, where 12.5% of the data was held out at a time from the models (Figure S7).

Species-specific mosquito abundance responses to land use

We investigated species-specific variation in responses to land use, by analysing the mean land use effects on species-level abundance for four *Aedes* and four *Anopheles* mosquito species that were most represented in the dataset (Table S6). We found that there was a high degree of divergence in the abundance responses of mosquito species to land use (Figure 3; Table S13). *Ae. aegypti* and *Ae. albopictus* diverged in their responses to substantial use intensity at primary vegetation sites, and at secondary vegetation sites (Figure 3). Whilst *Ae. aegypti* demonstrated a negative abundance response (-55%, 95% CI: -73.8% to -24.3%) at secondary vegetation sites, *Ae. albopictus* showed elevated abundance at both substantial use primary vegetation (94%, 95% CI: 26.1% – 198.9%) and secondary vegetation sites (68%, 95% CI: 4.7% - 167.3%). *Ae. aegypti* demonstrated the largest abundance response among the eight species, with a 207% increase in abundance at managed sites (95% CI: 75.7% - 435.4%), although there was a high degree of uncertainty associated with this estimate. Both *Ae. aegypti* and *Ae. albopictus* demonstrated an increased abundance trend at urban sites, although this was not significant. In contrast to *Ae. albopictus*, *Ae. scapularis* demonstrated reduced abundance in primary vegetation sites with substantial use intensity (-44%, 95% CI: -57.5% to -27.1%). Similarly, *Ae. serratus* abundance was reduced by 61% at primary vegetation sites with substantial use (95% CI: -75.5% to -36.6%) and by 66% in managed sites (95% CI: -79.3% to -44.5%).

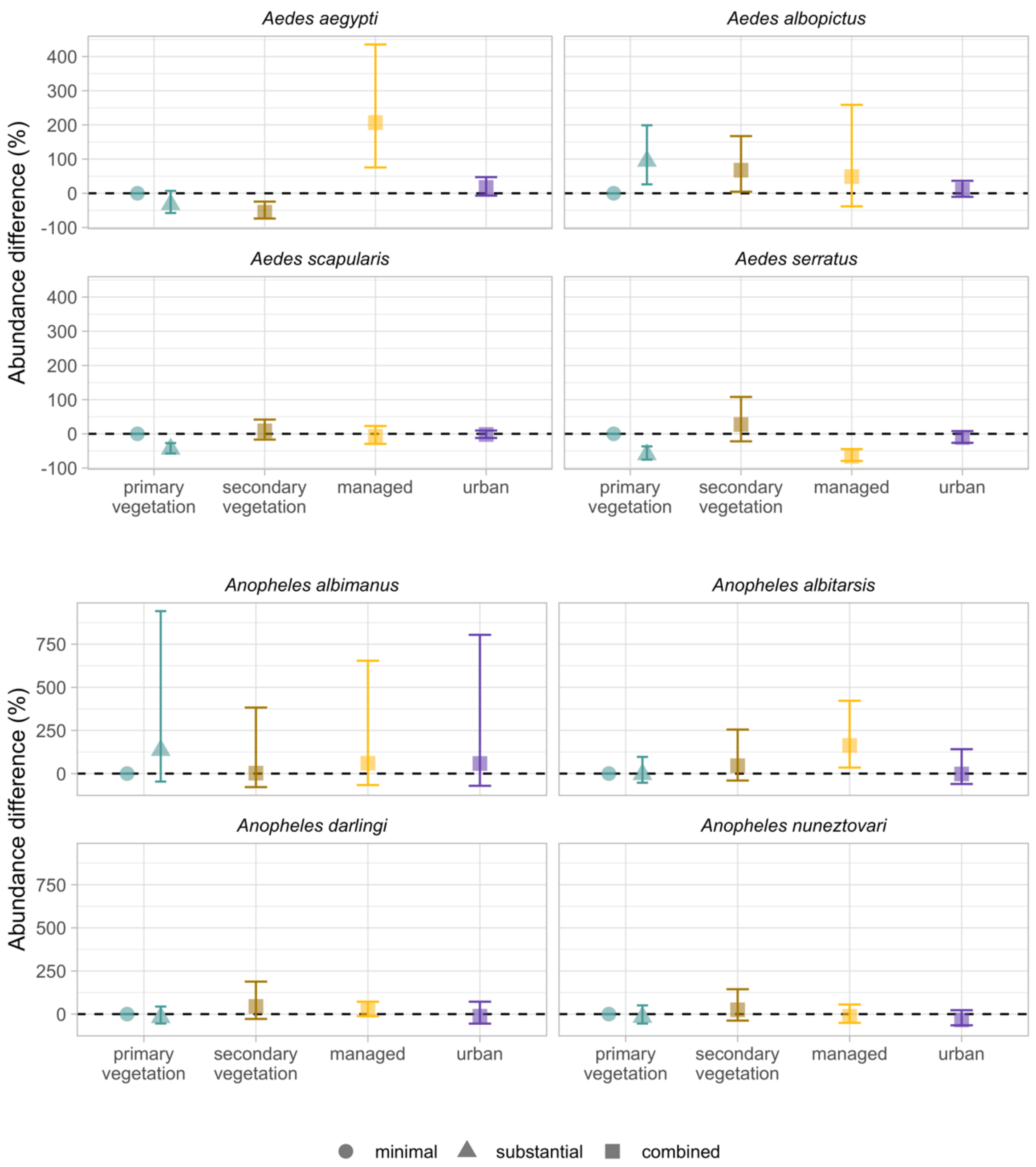

Figure 3. Species-specific mosquito abundance responses to land-use type and intensity. *Aedes* and *Anopheles* mosquito species abundance responses to land-use types with minimal (circles), substantial (triangles) and combined (squares) use intensities. For each genus, the four most represented species in the dataset were selected. Effect sizes were adjusted to a percentage by expressing each mean fixed effect and 95% credible intervals as a percentage of the baseline (primary vegetation minimal use, shown as zero). Intensity levels for secondary vegetation, managed and urban land uses were aggregated due to a lack of data representation.

The response of *Anopheles* mosquito abundance to different land uses in contrast to *Aedes* was less marked (Figure 3). Only the abundance of *An. albitarsis* mosquitoes was altered in comparison to the primary vegetation baseline. At managed sites, *An. albitarsis* abundance was 163% higher (95% CI: 34.8% - 422.2%). We detected a minimal impact of land use on *An. albimanus* and *An. nuneztovari* abundance and although the credible intervals crossed zero there was evidence of a trend towards higher *An. darlingi* abundance at secondary vegetation sites. There was a high level of uncertainty in the estimates for *An. albimanus,* possibly due to sparse sampling (only three urban, four managed, five secondary vegetation and ten primary vegetation sites). Holding out influential mosquito species records from genus-level abundance models overall did not markedly change the response to land use (Figure S8). However, models holding out *Ae. albopictus* records were sensitive to exclusion of data, as were estimates for managed land-use types.

Influence of deforestation on mosquito biodiversity

In contrast to spatial responses of mosquito biodiversity to land use, we found a strong temporal signal in the impact of deforestation on malaria mosquito species. Recent deforestation (in the last five years) was associated with higher *Anopheles* species richness (mean estimate 0.13, 95% CI: 0.03 - 0.23; Figure 4A). This result corresponded to a 14% increase in richness with every 1% increase in forest loss. Although not significant, there was also a trend towards increased abundance of *Anopheles* mosquitoes (Figure 4A). In contrast, we detected a minimal impact of deforestation on *Aedes* mosquito abundance and species richness. Furthermore, we found evidence of species-specific responses to deforestation. Whilst two *Anopheles* species responded positively to deforestation, there was a minimal impact of deforestation on the abundance of *Aedes* species (Figure 4B). *An. darlingi* demonstrated the largest increase in abundance with deforestation (mean estimate 0.28, 95% CI: 0.07 - 0.48; Figure 4B), followed by *An. albitarsis* (mean estimate 0.10, 95% CI: 0.05 - 0.16; Figure 4B). This corresponded to a 32% increase in *An. darlingi* abundance and an 11% increase for *An. albitarsis* with every 1% unit increase in forest loss.

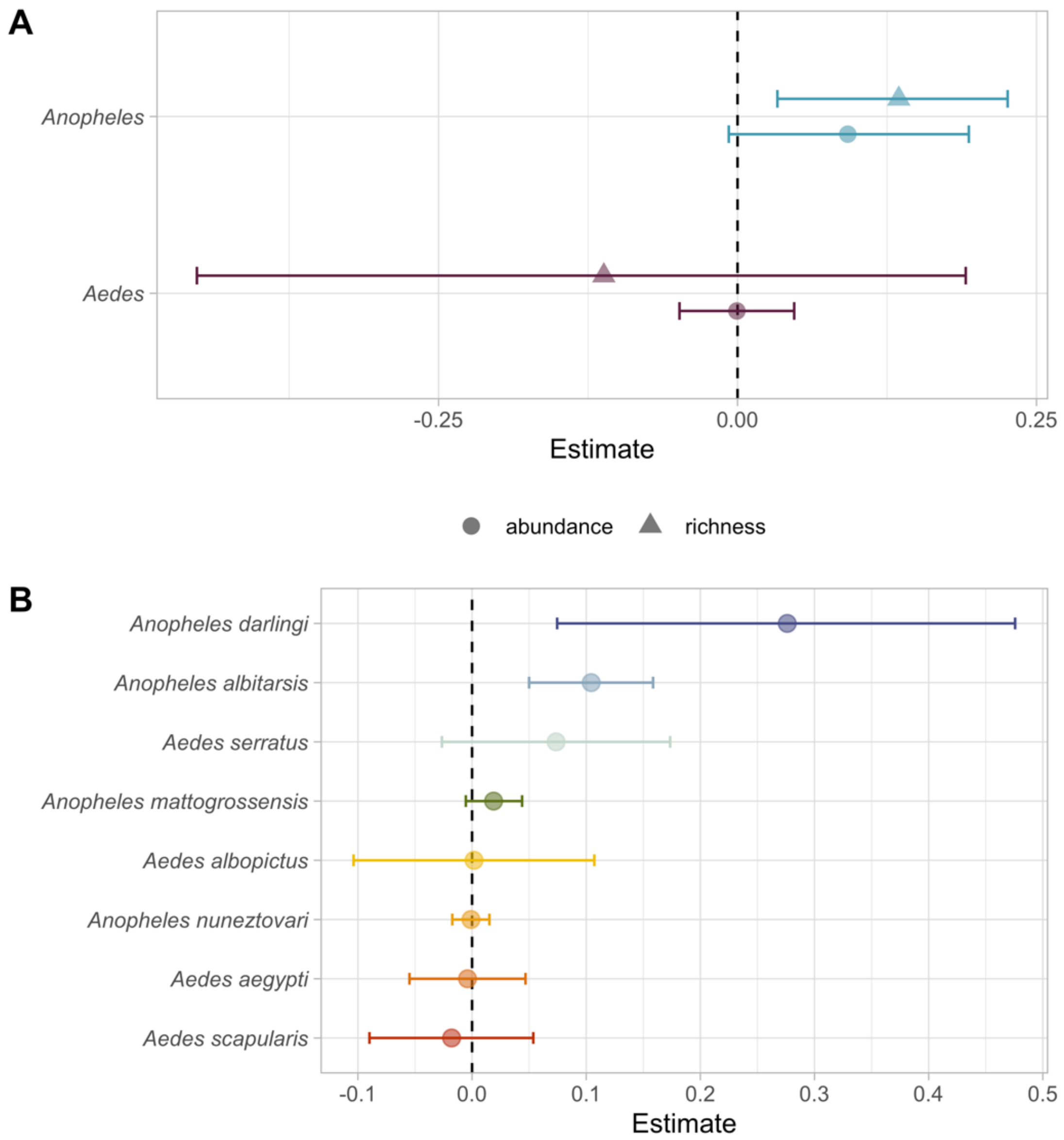

Figure 4. Impact of deforestation on mosquito species richness and abundance. Influence of recent deforestation on *Aedes* (purple) and *Anopheles* (blue) mosquito abundance (circles) and species richness (triangles; A). Abundance responses of eight mosquito species to recent deforestation (in the last five years; B). Points and bars for A and B show posterior mean and 95% credible intervals for linear fixed effects estimates of recent deforestation, calculated as proportional forest loss within the last five years of the sample start date for each site-level record.

Discussion

Our space-for-time approach provided an improved understanding of general responses of *Aedes* and *Anopheles* mosquitoes to land-use change. Overall, we found inconsistent responses of mosquito biodiversity to land use in Latin America and the Caribbean. However, both *Aedes*

and *Anopheles* mosquito species richness was reduced in urban environments and abundance of several synanthropic arbovirus (*Ae. aegypti* and *Ae. albopictus*) and malaria (*An. albitarsis*) vectors was greater in human-dominated landscapes. Further, we found a strong and consistent temporal signal of deforestation on malaria vector species. By integrating local landscape-level mosquito abundance records across 632 sites, we were able to detect substantial taxonomic differences in biodiversity responses to land use, providing a clear proof of concept for this methodology. Further, with the addition of more data these methods could be applied on a broader regional and global scale to investigate spatiotemporal dynamics of disease vector responses to environments subject to anthropogenic pressures.

Land use change is expected to lead to an overall decline in biodiversity, primarily due to habitat loss. However, disturbance can favour opportunistic species that are able to adapt to anthropogenic environments (20,48,49). The strong decrease in *Aedes* (26% reduction) and *Anopheles* (37% reduction) mosquito species richness in urban areas in this study is in agreement with previous studies that demonstrate decreased mosquito biodiversity in urban and fragmented landscapes (50,51). In some instances, biodiversity can provide a protective effect for disease emergence by regulating the abundance of vectors through intra- and interspecies competition, as well as through predation (52). Disruption of this protective effect can facilitate increased abundance of certain species able to adapt to novel environments. For example, decreased mosquito biodiversity in agricultural frontiers in the Amazon favours higher abundances of *An. darlingi* and drives subsequent malaria risk (53). Similarly in a malaria endemic region of Colombia, communities of *Anopheles* mosquitoes were less diverse in highly fragmented landscapes compared to more intact landscapes (51). In addition, we also found that deforestation was associated with increased *Anopheles* species richness, suggesting that the ecological changes caused by deforestation create novel habitats that increase abundance of select species (12,13).

In addition to genus-specific responses to land-use change, we additionally found pronounced species-specific abundance responses, highlighting the complexity in generalising mosquito responses to understand disease risk. The differential response of mosquito species to land-use change is likely to be driven by the unique life-history characteristics and habitat preferences of each species (54,55). We found increased abundance of opportunistic species in disturbed landscapes, including *Ae. aegypti* and *Ae. albopictus* mosquitoes. Several *Aedes* mosquitoes such as *Ae. aegypti* and *Ae. albopictus* are highly adapted to human-dominated landscapes,

which provide a range of artificial breeding sites such as crops, water storage containers, tyres and drains (21). These novel habitats facilitate increased abundance, densities, development and survival of *Aedes* mosquitoes in urban and agricultural areas (56,57). These findings have implications for the emergence of arboviruses transmitted by *Ae. aegypti* and *Ae. albopictus*, such as dengue, yellow fever and chikungunya, as disease transmission could be facilitated in anthropogenic environments without the implementation of adequate control measures. The strong response of these mosquitoes to anthropogenic disturbance combined with rapid development in the Amazon, expansion of arboviruses into urbanised forest (34) and rising climate suitability (58), poses a substantial threat of mosquito-borne disease in Latin America.

Whereas several *Aedes* mosquito species showed strong responses to land use, we detected a minimal response of *Anopheles* species. However, *An. albitarsis* an emerging secondary malaria vector in the Amazon (59,60) and the Atlantic Forest (61), exhibited higher abundances at managed sites. Furthermore, higher *An. albitarsis* abundance was also associated with increasing deforestation. This mosquito has become adapted to non-forest environments, where it exhibits highly anthropophilic behaviour (62,63). These results provide evidence that habitat alterations favouring secondary malaria vectors pose a substantial risk of disease emergence. In addition, the divergent abundance responses detected amongst *Aedes* and *Anopheles* mosquitoes highlights the importance of considering species-specific responses to land-use change when developing both predictive disease models and targeted control programs.

Anopheles

An. darlingi is a highly efficient anthropophilic malaria vector that predominates in the Amazon region (40,64). It has been well-documented that *An. darlingi* exhibits a preference for disturbed deforested landscapes, especially in locations close to human settlements in agricultural frontier regions (13,15). Here, *An. darlingi* exhibited the strongest response to deforestation and a trend towards increased abundance in secondary vegetation. This result is supported by previous findings demonstrating that *An. darlingi* is favoured by deforestation (12,13). Secondary growth, particularly at forest fringes, offers a range of suitable environmental conditions for *An. darlingi*, including increased sunlight, refugia and ground pools (15,65,66). However, here we could not detect a significant abundance response of *An. darlingi* at secondary vegetation sites, with only two mosquito species demonstrating significant responses to recent deforestation. These results suggest that the relationship between deforestation and mosquito-borne disease risk is inherently complex and may be

dependent on fine-scale factors, such as microclimatic variation and predation (67,68) and that cannot easily be summarised over a broad scale.

Despite providing evidence of species- and genus-specific responses to land-use change, this study has several limitations. First, owing to the high levels of mosquito biodiversity in the Amazon and Atlantic forests captured in this study, the dataset is geographically biased towards these regions and rainforest biomes. However, the findings of this study were broadly robust to both random and species-level subsampling. Second, studies included in the dataset are likely to underestimate the true abundance of mosquito species as the methods employed in each study were biased towards anthropophilic mosquitoes and species that are easier to find and capture. Nonetheless, several studies included in the dataset sampled mosquitoes using multiple sampling methods. For example, mosquito sampling was performed in many studies using human-landing catch, which primarily captures anthropophilic mosquitoes, although can be used to capture both endophilic (indoor-resting) and exophilic (outdoor) mosquitoes (69). Other studies used baited traps and ovitraps left overnight to capture nocturnal mosquitoes and those at different life stages, such as larvae and pupae.

In addition, the random effects structure of the model accounted for differing sampling methodologies that may explain a proportion of the variation in mosquito abundance observed. The mosquito species included in the dataset are likely to be biased towards dominant and incriminated vector species, such as *An. darlingi* and *Ae. aegypti*. Future assessments could consider species bias by taking into account publication effort (8) to ensure under-represented species that may well be efficient vectors of human diseases are included. Additionally, abundance records in the dataset included a high number of zero observations, resulting from sampling of species at sites where occurrence was low. Incorporating species occurrence probability into the modelling framework may be a method to address the zero-inflation of abundance data (8).

Ecological changes caused by anthropogenic land-use change have a wide range of cascading effects on mosquito-borne disease risk. A comprehensive grasp of how mosquito species are affected by anthropogenic disturbance will facilitate the development of highly effective disease control measures. A greater understanding could additionally equip vector control efforts with species-specific information to support targeted elimination efforts for mosquito-borne diseases such as dengue, yellow fever, malaria and chikungunya. This study has

presented a comparative dataset of 10,004 *Aedes* and *Anopheles* mosquito records in Latin America and the Caribbean, which is a valuable resource for investigating the effect of land-use change on mosquito-borne disease risk that is epidemiologically relevant at the regional scale. We demonstrate considerable species-specific responses, which represent the diverging impacts of land-use change on mosquito fauna and caution against generalising predictions of vector responses to environmental change. These findings add to the knowledge base of how opportunistic species contribute to elevated mosquito-borne disease risk in anthropogenic environments.

Make sure that species names are properly italicized in the references

References

1. Popp A, Calvin K, Fujimori S, Havlik P, Humpenöder F, Stehfest E, et al. Land-use futures in the shared socio-economic pathways. Glob Environ Chang [Internet]. 2017;42:331–45. Available from: https://www.sciencedirect.com/science/article/pii/S0959378016303399
2. Winkler K, Fuchs R, Rounsevell M, Herold M. Global land use changes are four times greater than previously estimated. Nat Commun [Internet]. 2021;12(1):2501. Available from: https://doi.org/10.1038/s41467-021-22702-2
3. Arneth A, Denton F, Agus F, Elbehri A, Erb K, Osman Elasha B, et al. Framing and Context. In: Shukla PR, Skea J, Buendia EC, Masson-Delmotte V, Pörtner H-O, Roberts DC, et al., editors. Climate Change and Land: An IPCC Special Report on Climate Change, Desertification, Land Degradation, Sustainable Land Management, Food Security, and Greenhouse Gas Fluxes in Terrestrial Ecosystems. 2019.
4. Newbold T, Hudson LN, Hill SLLL, Contu S, Lysenko I, Senior RA, et al. Global effects of land use on local terrestrial biodiversity. Nature. 2015 Apr 1;520(7545):45–50.
5. Daszak P, Cunningham AA, Hyatt AD. Anthropogenic environmental change and the emergence of infectious diseases in wildlife. Acta Trop. 2001 Feb;78(2):103–16.
6. Keesing F, Belden LK, Daszak P, Dobson A, Harvell CD, Holt RD, et al. Impacts of biodiversity on the emergence and transmission of infectious diseases. Nature [Internet]. 2010;468(7324):647–52. Available from: http://dx.doi.org/10.1038/nature09575
7. Gottdenker NL, Streicker DG, Faust CL, Carroll CR. Anthropogenic land use change and infectious diseases: a review of the evidence. Ecohealth. 2014 Dec;11(4):619–32.
8. Gibb R, Redding DW, Chin KQ, Donnelly CA, Blackburn TM, Newbold T, et al. Zoonotic host diversity increases in human-dominated ecosystems. Nature [Internet]. 2020;584(7821):398–402. Available from: https://doi.org/10.1038/s41586-020-2562-8
9. IPCC. Climate Change 2021: The Physical Science Basis. Contribution of Working Group I to the Sixth Assessment Report of the Intergovernmental Panel on Climate Change. Masson-Delmotte V, Zhai P, Pirani A, Connors SL, Péan C, Berger S, et al., editors. Cambridge University Press. In Press.; 2021.
10. Bhatt S, Gething PW, Brady OJ, Messina JP, Farlow AW, Moyes CL, et al. The global distribution and burden of dengue. Nature [Internet]. 2013;496(7446):504–7. Available from: https://www.scopus.com/inward/record.uri?eid=2-s2.0-84876804736&doi=10.1038%2Fnature12060&partnerID=40&md5=b4f9e76203e38a8874c74e04460587c7
11. Fletcher IK, Grillet ME, Moreno JE, Drakeley C, Hernández-Villena J, Jones KE, et al. Synergies between environmental degradation and climate variation on malaria re-emergence in southern Venezuela: a spatiotemporal modelling study. Lancet Planet Heal [Internet]. 2022 Sep 1;6(9):e739–48. Available from: https://doi.org/10.1016/S2542-5196(22)00192-9
12. Burkett-Cadena ND, Vittor AY. Deforestation and vector-borne disease: Forest conversion favors important mosquito vectors of human pathogens. Basic Appl Ecol. 2018;26:101–10.
13. Vittor AY, Pan W, Gilman RH, Tielsch J, Glass G, Shields T, et al. Linking Deforestation to

Malaria in the Amazon: Characterization of the Breeding Habitat of the Principal Malaria Vector, Anopheles darlingi. Am J Trop Med Hyg. 2009 Jul;81(1):5–12.
14. Vittor AY, Gilman RH, Tielsch JM, Glass G, Shields T, Sanchez-Lozano W, et al. The effect of deforestation on the human-biting rate of Anopheles darlingi, the primary vector of Falciparum malaria in the Peruvian Amazon. Am J Trop Med Hyg. 2006 Dec;74(1):3–11.
15. Barros F, Arruda M, Gurgel H, Honório N. Spatial clustering and longitudinal variation of Anopheles darlingi (Diptera: Culicidae) larvae in a river of the Amazon: the importance of the forest fringe and of obstructions to flow in frontier malaria. Bull Entomol Res. 2011 Dec;101(6):643–58.
16. Laporta GZ, Ilacqua RC, Bergo ES, Chaves LSM, Rodovalho SR, Moresco GG, et al. Malaria transmission in landscapes with varying deforestation levels and timelines in the Amazon: a longitudinal spatiotemporal study. Sci Rep [Internet]. 2021;11(1):6477. Available from: https://doi.org/10.1038/s41598-021-85890-3
17. Oliveira TMP, Laporta GZ, Bergo ES, Chaves LSM, Antunes JLF, Bickersmith SA, et al. Vector role and human biting activity of Anophelinae mosquitoes in different landscapes in the Brazilian Amazon. Parasit Vectors [Internet]. 2021;14(1):236. Available from: http://ovidsp.ovid.com/ovidweb.cgi?T=JS&PAGE=reference&D=prem&NEWS=N&AN=33957959
18. de Castro MC, Monte-Mór RL, Sawyer DO, Singer BH. Malaria risk on the Amazon frontier. Proc Natl Acad Sci [Internet]. 2006 Feb 14;103(7):2452–7. Available from: http://www.pnas.org/content/103/7/2452.abstract
19. Harbach R. Mosquito Taxonomic Inventory. Valid Species List [Internet]. 2020. Available from: http://mosquito-taxonomic-inventory.info/valid-species-list
20. Wilke ABB, Benelli G, Beier JC. Anthropogenic changes and associated impacts on vector-borne diseases. Trends Parasitol [Internet]. 2021;37(12):1027–30. Available from: https://www.sciencedirect.com/science/article/pii/S1471492221002385
21. Paploski IAD, Rodrigues MS, Mugabe VA, Kikuti M, Tavares AS, Reis MG, et al. Storm drains as larval development and adult resting sites for Aedes aegypti and Aedes albopictus in Salvador, Brazil. Parasites and Vectors [Internet]. 2016;9(1):419. Available from: https://www.scopus.com/inward/record.uri?eid=2-s2.0-84979524694&doi=10.1186%2Fs13071-016-1705-0&partnerID=40&md5=1a2d392db759843ce8af5daf411b5771
22. de Carvalho GC, Ceretti-Junior W, Barrio-Nuevo KM, Wilk-da-Silva R, Christe RO, de Paula MB, et al. Composition and diversity of mosquitoes (Diptera: Culicidae) in urban parks in the South region of the city of Sao Paulo, Brazil. Biota Neotrop. 2017;17(2).
23. Wilke ABB, Chase C, Vasquez C, Carvajal A, Medina J, Petrie WD, et al. Urbanization creates diverse aquatic habitats for immature mosquitoes in urban areas. Sci Rep [Internet]. 2019;9(1):15335. Available from: https://doi.org/10.1038/s41598-019-51787-5
24. Wilke ABB, Vasquez C, Carvajal A, Medina J, Chase C, Cardenas G, et al. Proliferation of Aedes aegypti in urban environments mediated by the availability of key aquatic habitats. Sci Rep [Internet]. 2020;10(1):12925. Available from: https://doi.org/10.1038/s41598-020-69759-5
25. Sinka ME, Pironon S, Massey NC, Longbottom J, Hemingway J, Moyes CL, et al. A new malaria vector in Africa: Predicting the expansion range of Anopheles stephensi and identifying the urban populations at risk. Proc Natl Acad Sci U S A. 2020 Oct;117(40):24900–8.
26. Câmara DCP, Pinel C da S, Rocha GP, Codeço CT, Honório NA, Camara DCP, et al. Diversity of mosquito (Diptera: Culicidae) vectors in a heterogeneous landscape endemic for arboviruses. Acta Trop [Internet]. 2020;212:105715. Available from: http://ovidsp.ovid.com/ovidweb.cgi?T=JS&PAGE=reference&D=medl&NEWS=N&AN=32971068
27. Loaiza JR, Dutari LC, Rovira JR, Sanjur OI, Laporta GZ, Pecor J, et al. Disturbance and mosquito diversity in the lowland tropical rainforest of central Panama. Sci Rep [Internet]. 2017;7(1):7248. Available from: https://doi.org/10.1038/s41598-017-07476-2
28. Abella-Medrano CA, Ibanez-Bernal S, MacGregor-Fors I, Santiago-Alarcon D.

Spatiotemporal variation of mosquito diversity (Diptera: Culicidae) at places with different land-use types within a neotropical montane cloud forest matrix. Parasit Vectors. 2015;8:487.

29. Reiskind MH, Griffin RH, Janairo MS, Hopperstad KA. Mosquitoes of field and forest: the scale of habitat segregation in a diverse mosquito assemblage. Med Vet Entomol. 2017 Mar;31(1):44–54.

30. Du M, Jing W, Liu M, Liu J. The global trends and regional differences in incidence of dengue infection from 1990 to 2019: An analysis from the Global Burden of Disease Study 2019. Infect Dis Ther [Internet]. 2021;10(3):1625–43. Available from: https://doi.org/10.1007/s40121-021-00470-2

31. PAHO. 10 vector-borne diseases that put the population of the Americas at risk [Internet]. 2021 [cited 2021 Oct 28]. Available from: https://www3.paho.org/hq/index.php?option=com_content&view=article&id=9438:2014-10-vector-borne-diseases-that-put-population-americas-at-risk&Itemid=135&lang=en

32. Vasconcelos PF, Travassos da Rosa AP, Rodrigues SG, Travassos da Rosa ES, Degallier N, Travassos da Rosa JF. Inadequate management of natural ecosystem in the Brazilian Amazon region results in the emergence and reemergence of arboviruses. Cad Saude Publica. 2001;17 (Suppl.:155–64.

33. Macedo MN, DeFries RS, Morton DC, Stickler CM, Galford GL, Shimabukuro YE. Decoupling of deforestation and soy production in the southern Amazon during the late 2000s. Proc Natl Acad Sci [Internet]. 2012 Jan 24;109(4):1341–6. Available from: http://www.pnas.org/content/109/4/1341.abstract

34. Lowe R, Lee S, Martins Lana R, Torres Codeço C, Castro MC, Pascual M. Emerging arboviruses in the urbanized Amazon rainforest. BMJ. 2020;371:m4385.

35. Wilcox B, Ellis B. Forests and emerging infectious diseases of humans. Unasylva. 2006 Jan 1;57:11–8.

36. Grillet ME, Moreno JE, Hernández-Villena J V, Vincenti-González MF, Noya O, Tami A, et al. Malaria in Southern Venezuela: The hottest hotspot in Latin America. PLoS Negl Trop Dis. 2021 Jan 25;15(1):e0008211.

37. Hudson LN, Newbold T, Contu S, Hill SLL, Lysenko I, De Palma A, et al. The PREDICTS database: a global database of how local terrestrial biodiversity responds to human impacts. Ecol Evol. 2014 Dec;4(24):4701–35.

38. De Palma A, Abrahamczyk S, Aizen MA, Albrecht M, Basset Y, Bates A, et al. Predicting bee community responses to land-use changes: Effects of geographic and taxonomic biases. Sci Rep [Internet]. 2016;6(1):31153. Available from: http://www.nature.com/articles/srep31153

39. Millard J, Outhwaite CL, Kinnersley R, Freeman R, Gregory RD, Adedoja O, et al. Global effects of land-use intensity on local pollinator biodiversity. Nat Commun [Internet]. 2021;12(1):2902. Available from: https://doi.org/10.1038/s41467-021-23228-3

40. Sinka ME, Rubio-Palis Y, Manguin S, Patil AP, Temperley WH, Gething PW, et al. The dominant Anopheles vectors of human malaria in the Americas: occurrence data, distribution maps and bionomic précis. Parasit Vectors [Internet]. 2010;3(1):72. Available from: https://doi.org/10.1186/1756-3305-3-72

41. Kraemer MUG, Sinka ME, Duda KA, Mylne AQN, Shearer FM, Barker CM, et al. The global distribution of the arbovirus vectors Aedes aegypti and Ae. albopictus. Elife. 2015 Jun;4:e08347.

42. WWF. Terrestrial Ecoregions [Internet]. 2021 [cited 2021 Jun 15]. Available from: https://www.worldwildlife.org/biome-categories/terrestrial-ecoregions

43. Spiegelhalter DJ, Best NG, Carlin BP, Van Der Linde A. Bayesian measures of model complexity and fit. J R Stat Soc Ser B (Statistical Methodol [Internet]. 2002 Oct 1;64(4):583–639. Available from: https://doi.org/10.1111/1467-9868.00353

44. Watanabe S. Asymptotic Equivalence of Bayes Cross Validation and Widely Applicable Information Criterion in Singular Learning Theory. J Mach Learn Res. 2010 Dec;11(116):3571–3594.

45. Blangiardo M, Cameletti M, Baio G, Rue H. Spatial and spatio-temporal models with R-INLA. Spat Spatiotemporal Epidemiol. 2013 Dec;7:39–55.

46. Hansen M., Potapov P., Moore R, Hancher M, Turubanova S., Tyukavina A, et al. High-

Resolution Global Maps of 21st-Century Forest Cover Change. Science (80-) [Internet]. 2013 Nov 15;342(6160):850–3. Available from: http://science.sciencemag.org/content/342/6160/850.abstract

47. Verdonschot PFM, Besse-Lototskaya AA. Flight distance of mosquitoes (Culicidae): A metadata analysis to support the management of barrier zones around rewetted and newly constructed wetlands. Limnologica [Internet]. 2014;45:69–79. Available from: https://www.sciencedirect.com/science/article/pii/S0075951113001011

48. McKinney ML. Urbanization, Biodiversity, and Conservation: The impacts of urbanization on native species are poorly studied, but educating a highly urbanized human population about these impacts can greatly improve species conservation in all ecosystems. Bioscience [Internet]. 2002 Oct 1;52(10):883–90. Available from: https://doi.org/10.1641/0006-3568(2002)052[0883:UBAC]2.0.CO

49. Multini LC, Marrelli MT, Beier JC, Wilke ABB. Increasing Complexity Threatens the Elimination of Extra-Amazonian Malaria in Brazil. Trends Parasitol [Internet]. 2019;35(6):383–7. Available from: https://www.scopus.com/inward/record.uri?eid=2-s2.0-85064321601&doi=10.1016%2Fj.pt.2019.03.009&partnerID=40&md5=6d66c9cf36dd524e615d4084e6a52ae2

50. Ferraguti M, Martínez-de la Puente J, Roiz D, Ruiz S, Soriguer R, Figuerola J. Effects of landscape anthropization on mosquito community composition and abundance. Sci Rep [Internet]. 2016 Jul 4;6:29002. Available from: https://doi.org/10.1038/srep29002

51. Hernández-Valencia JC, Rincón DS, Marín A, Naranjo-Díaz N, Correa MM. Effect of land cover and landscape fragmentation on anopheline mosquito abundance and diversity in an important Colombian malaria endemic region. PLoS One [Internet]. 2020 Oct 15;15(10):e0240207. Available from: https://doi.org/10.1371/journal.pone.0240207

52. Laporta GZ, Lopez de Prado PIK, Kraenkel RA, Coutinho RM, Sallum MAM. Biodiversity can help prevent malaria outbreaks in tropical forests. PLoS Negl Trop Dis. 2013;7(3):e2139.

53. Chaves LSM, Bergo ES, Conn JE, Laporta GZ, Prist PR, Sallum MAM. Anthropogenic landscape decreases mosquito biodiversity and drives malaria vector proliferation in the Amazon rainforest. PLoS One. 2021;16(1):e0245087.

54. Becker N, Petric D, Zgomba M, Boase C, Madon MB, Dahl C, et al. Mosquitoes and their control. 2nd ed. Berlin, Germany: Springer Verlag; 2010.

55. Tucker Lima JM, Vittor A, Rifai S, Valle D. Does deforestation promote or inhibit malaria transmission in the Amazon? A systematic literature review and critical appraisal of current evidence. Philos Trans R Soc Lond B Biol Sci. 2017/04/24. 2017 Jun;372(1722):20160125.

56. Li Y, Kamara F, Zhou G, Puthiyakunnon S, Li C, Liu Y, et al. Urbanization increases Aedes albopictus larval habitats and accelerates mosquito development and survivorship. PLoS Negl Trop Dis. 2014 Nov;8(11):e3301.

57. Thongsripong P, Green A, Kittayapong P, Kapan D, Wilcox B, Bennett S. Mosquito Vector Diversity across Habitats in Central Thailand Endemic for Dengue and Other Arthropod-Borne Diseases. PLoS Negl Trop Dis [Internet]. 2013 Oct 31;7(10):e2507. Available from: https://doi.org/10.1371/journal.pntd.0002507

58. Romanello M, McGushin A, Di Napoli C, Drummond P, Hughes N, Jamart L, et al. The 2021 report of the Lancet Countdown on health and climate change: code red for a healthy future. Lancet. 2021 Oct 21;398(10311):1619–62.

59. Povoa MM, de Souza RTL, Lacerda RN da L, Rosa ES, Galiza D, de Souza JR, et al. The importance of Anopheles albitarsis E and An. darlingi in human malaria transmission in Boa Vista, state of Roraima, Brazil. Mem Inst Oswaldo Cruz. 2006 Mar;101(2):163–8.

60. Abou Orm S, Moreno JE, Carrozza M, Acevedo P, Herrera F. Plasmodium spp. infection rates for some Anopheles spp. from Sifontes Municipality, Bolívar State, Venezuela. Bol Malariol y Salud Ambient. 2017;57(1):17–24.

61. da Silva KS, Pinto I de S, Leite GR, das Virgens TM, dos Santos CB, Falqueto AA, et al. Ecology of Anopheline Mosquitoes (Diptera: Culicidae) in the Central Atlantic Forest Biodiversity Corridor, Southeastern Brazil. J Med Entomol. 2013 Jan 22;50(1):24–30.

62. Foley DH, Linton Y-M, Freddy Ruiz-Lopez J, Conn JE, Sallum MAM, Povoa MM, et al. Geographic distribution, evolution, and disease importance of species within the Neotropical

Anopheles albitarsis Group (Diptera, Culicidae). J Vector Ecol. 2014 Jun;39(1):168–81.
63. Tadei WP, Dutary Thatcher B. Malaria vectors in the Brazilian Amazon: Anopheles of the subgenus Nyssorhynchus. Vol. 42, Revista do Instituto de Medicina Tropical de São Paulo. 2000. p. 87–94.
64. Natal D, Urbinatti PR, Malafronte RDS, Rezende HR, Cerutti Jr. C, Sallum MAM. First record of Anopheles (Anopheles) costai Fonseca & Ramos, 1939 in Espírito Santo State, Brazil. Rev Inst Med Trop Sao Paulo [Internet]. 2007;49(5):323–6. Available from: https://www.scopus.com/inward/record.uri?eid=2-s2.0-36248983784&doi=10.1590%2FS0036-46652007000500009&partnerID=40&md5=cc863ddc34d56eda6562097ae5604b02
65. Rejmánková E, Grieco J, Achee N, Roberts DR. Ecology of Larval Habitats. In: Anopheles Mosquitoes - New insights into malaria vectors [Internet]. Sylvie Manguin, IntechOpen; 2013. Available from: https://www.intechopen.com/chapters/43671
66. Sanchez-Ribas J, Oliveira-Ferreira J, Gimnig JE, Pereira-Ribeiro C, Alberto Santos-Neves MS, Silva-do-Nascimento TF, et al. Environmental variables associated with anopheline larvae distribution and abundance in Yanomami villages within unaltered areas of the Brazilian Amazon. Parasit Vectors. 2017 Nov;10(1):571.
67. Kweka EJ, Kimaro EE, Munga S. Effect of Deforestation and Land Use Changes on Mosquito Productivity and Development in Western Kenya Highlands: Implication for Malaria Risk. Front Public Heal. 2016;4(238).
68. Afrane YA, Zhou G, Lawson BW, Githeko AK, Yan G. Effects of microclimatic changes caused by deforestation on the survivorship and reproductive fitness of Anopheles gambiae in western Kenya highlands. Am J Trop Med Hyg. 2006 May;74(5):772–8.
69. Lima JBP, Rosa-Freitas MG, Rodovalho CM, Santos F, Lourenço-de-Oliveira R. Is there an efficient trap or collection method for sampling Anopheles darlingi and other malaria vectors that can describe the essential parameters affecting transmission dynamics as effectively as human landing catches? - A review. Mem Inst Oswaldo Cruz [Internet]. 2014;109(5):685–705. Available from: https://www.scopus.com/inward/record.uri?eid=2-s2.0-84906739342&doi=10.1590%2F0074-0276140134&partnerID=40&md5=dea2ef1aa0a7f58d57e535fbeba4e663

Acknowledgements: Special thanks to members of the Planetary Health & Infectious Disease (PHID) Lab at for their support and the Kate Jones' lab at UCL for informal feedback on this work.

Author contributions: IKF, KJ and RL conceived the study. IKF collated the data, fitted the models with assistance from RG, IKF analysed the results and wrote the manuscript. RG, RL and KJ discussed the results and provided interpretation. RG, RL reviewed and revised the manuscript, all authors approved the final version of the manuscript.

Funding: IKF was supported by the Biological and Biotechnology Research Council (grant BB/M009513/1) and RL was supported by a Royal Society Dorothy Hodgkin fellowship. RG was supported by a Graduate Research Fellowship from University College London.

Declaration of interests: The authors declare no conflicts of interest.

Data accessibility statement: All data and code that support the findings of this study are available in the Github repository https://github.com/isabelfletcher/vectors_landuse

Figure1 Click here to access/download;Figure;figure1

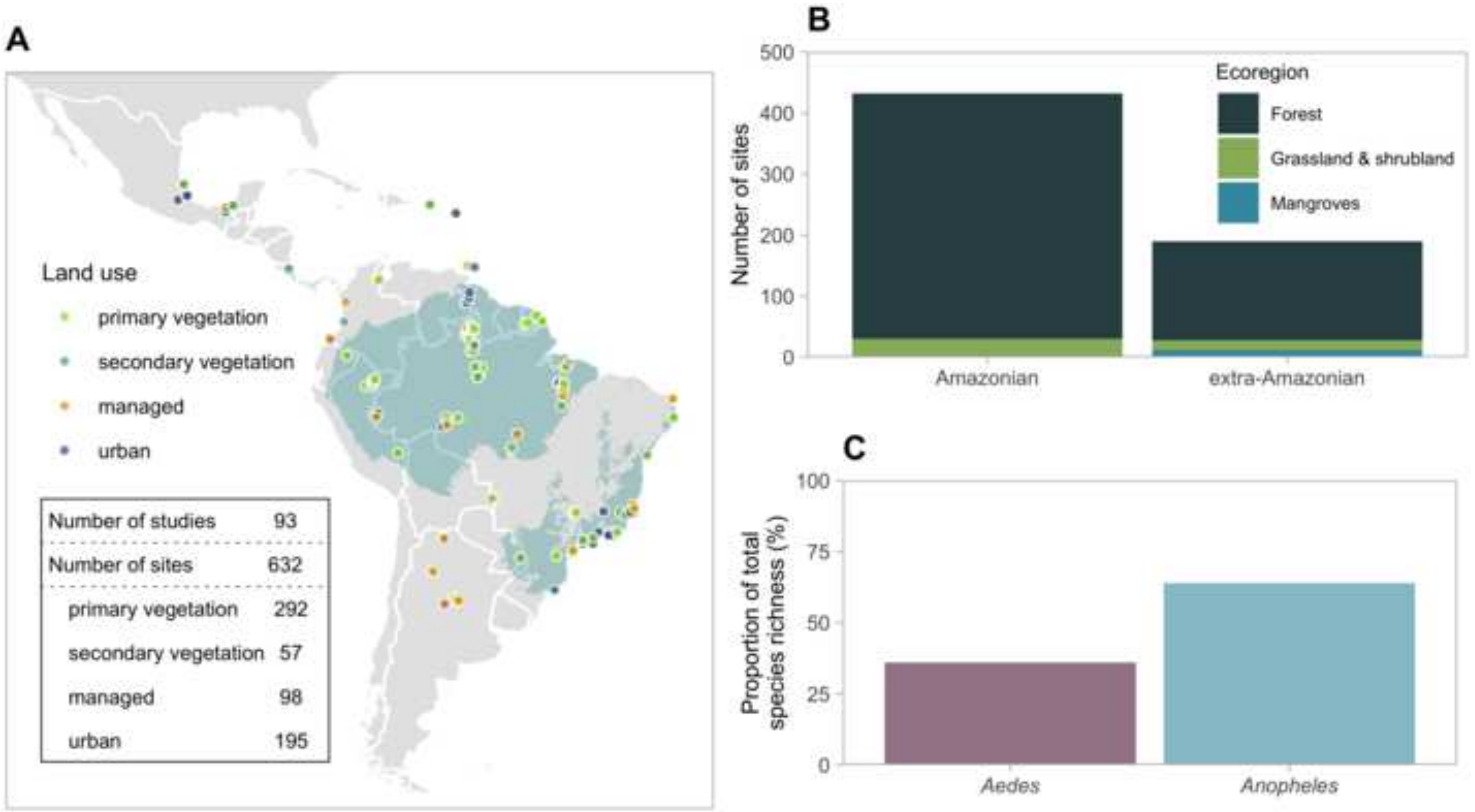

Figure2

Click here to access/download;Figure;figure2

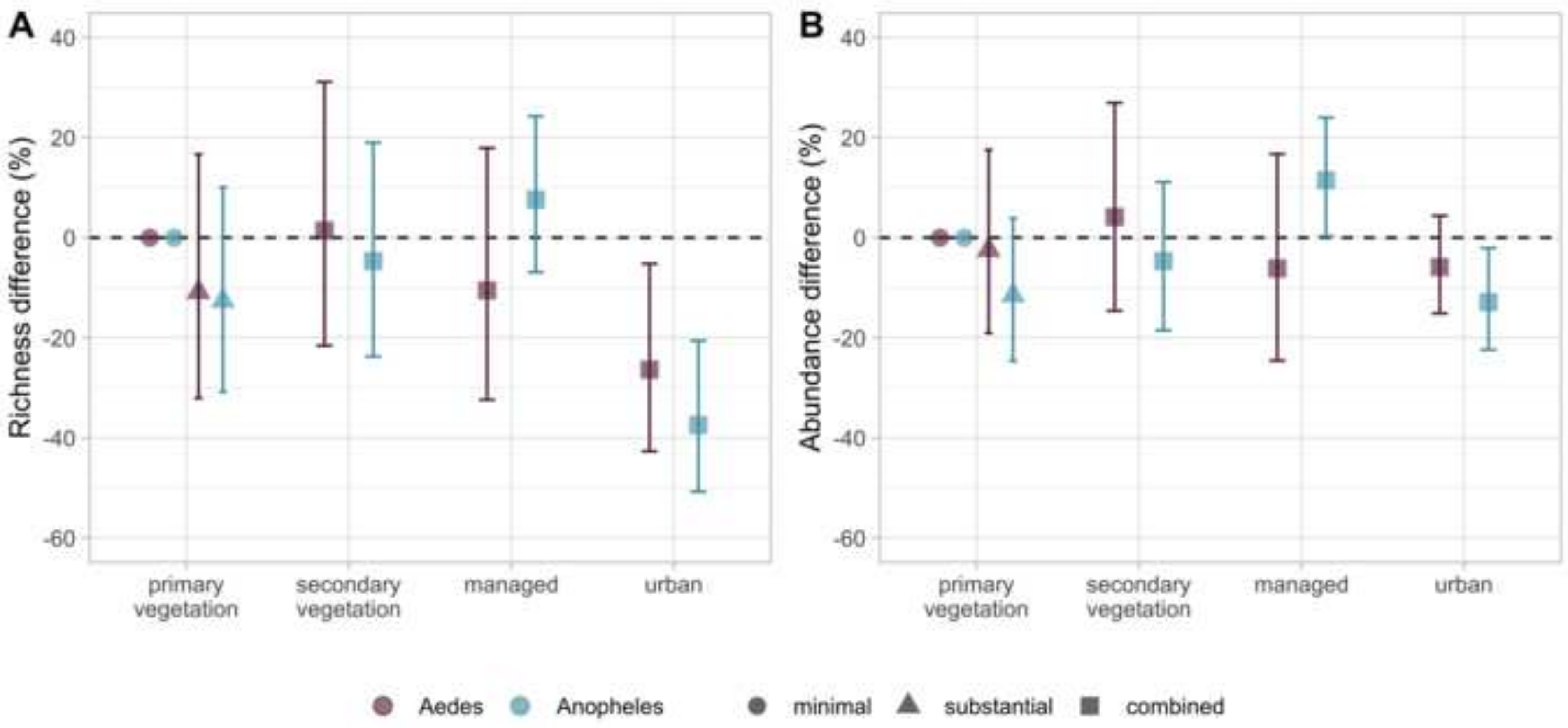

 Click here to access/download;Figure;figure3

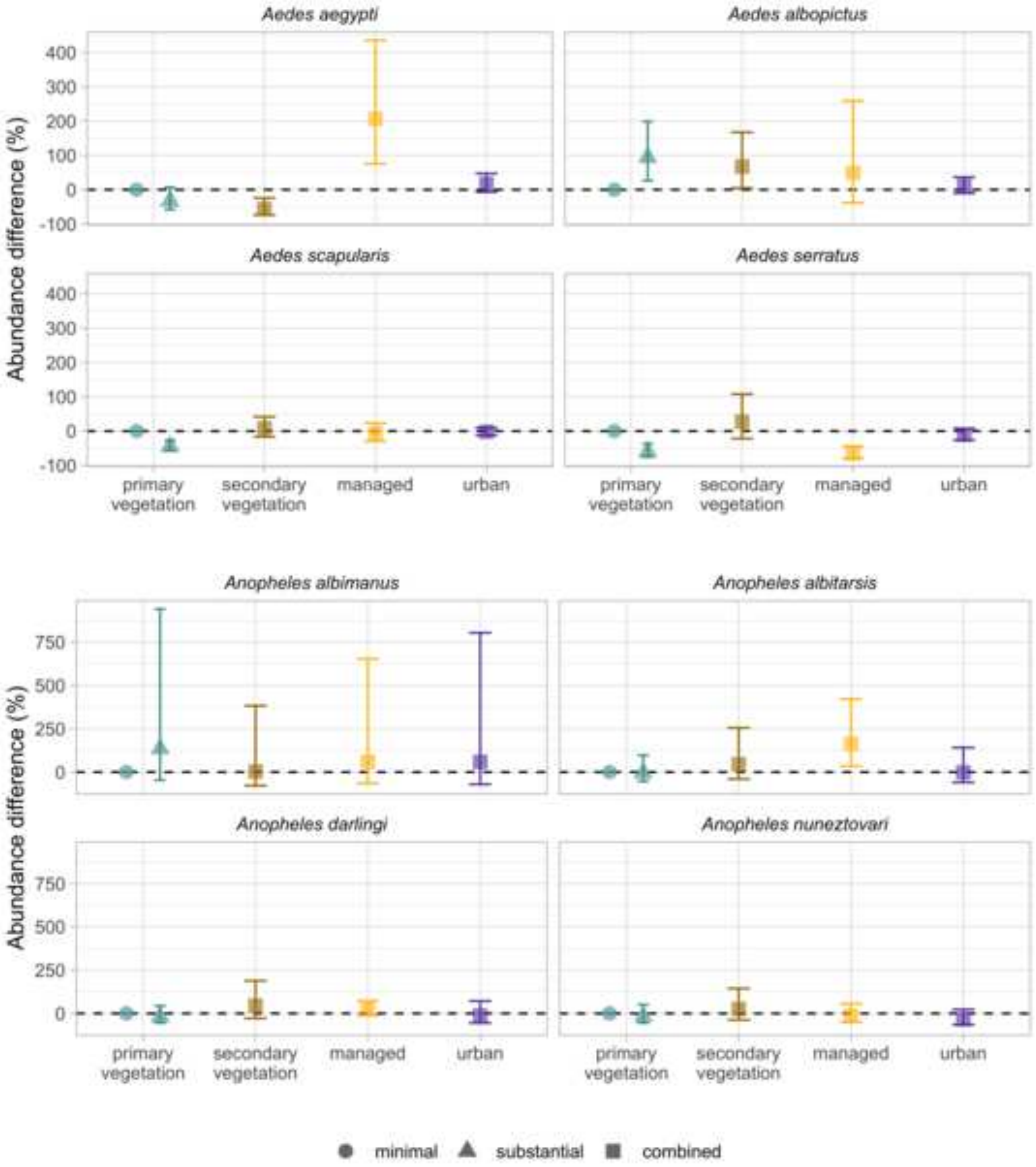

Figure4

Click here to access/download;Figure;figure4

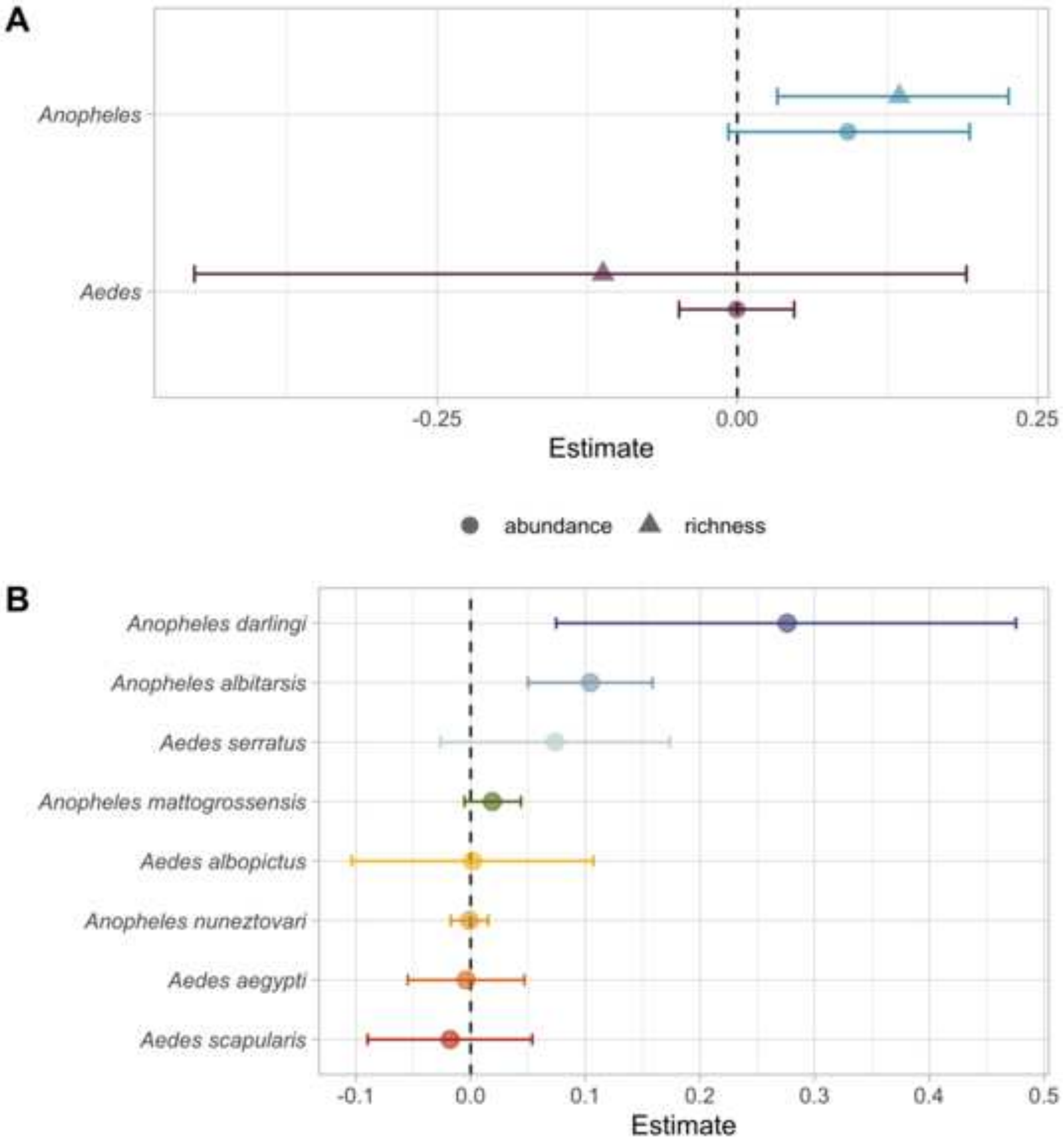

Click here to access/download
Supporting Information
supplementary_IF.docx
