# Supplementary material for: Differing taxonomic responses of mosquito vectors to anthropogenic land-use change in Latin America and the Caribbean
Source: PLoS Negl Trop Dis. 2023 Jul 14;17(7):e0011450. doi: 10.1371/journal.pntd.0011450 (PMC10348580; doi:10.1371/journal.pntd.0011450)
Supplement: Attachment — Submitted filename: Review_AF.docx [file pntd.0011450.s025.docx]

This is a very interesting study investigating the impact of land-use on mosquito vectors abundance and richness at a regional scale. Firstly, I would like to congratulate the authors on piecing together such an extensive dataset. Having done a few big data scrapping myself, I can only appreciate the challenge of collecting data from multiple sources to create such a thing. Overall, I thoroughly enjoyed reading this manuscript: it is well written (with few grammatical errors), the statistical framework is well defined, solid and well explained, the logic is well followed through the manuscript, and it addresses a key issue in landscape disease ecology. I do have a few comments that I hope will help improving the manuscript, or maybe just give the authors food for thoughts.

The main issue I have with the manuscript in it’s current form is related to the statistical analysis. The authors investigate land use and their respective effects on mosquito richness and abundance across 4 categories (primary vegetation, secondary vegetation, managed and urban), however, I believe that a more solid approach related to urban gradients and land-use would be to use a continuous variable rather than an assigned category. For instance, using the APPEARS resource on EarthData would allow the authors to use more adequate proxies of land-use, such as EVI or nightlight rasters, thus perhaps giving a different perspective on their results. This is but food for thoughts, as I believe the current analysis to have been done appropriately.

There is also a seemingly puzzling fact: looking at figure 2 and 3, there seem to be a decrease in both abundance and richness of mosquitoes in urban areas. Keeping in mind that these data were analysed as categories, I’m wondering why the authors found a positive abundance response to urbanisation in figure 4. I understand that figure 4 relates to changes in land use over time as opposed to assigned land-usage, but still this change in effect directionality seems unlikely. Again, this relates to my previous comment, and I’m wondering if using a continuous, satellite-based index would change the directionality of the effect shown in figure 2 and 3.

Lastly, I have but a curiosity comment: since the authors are working on a multi-species system, would it be possible to create a phylogenetic tree of the mosquito species present in the collected database in order to include a phylogenetic signal in the analyses? I would not be too surprised to see recent evolutionary history playing a major role in shaping abundance and richness pattern linked with land-usage at a species level. Again, this is but food for thoughts.

Minor comments:

Line 57: Global warming?

Line 71: I am not sure this statement is true; I can think of a few studies that were published in the past few years specifically focused on large-scale mosquito patterns (see Steiger et al. 2016, Rakotoarinia et al. 2022), and even more so on the amazon basin (MacDonald and Mordecal 2019).

Line 78: I would argue that it negatively impacts native biodiversity, not biodiversity as a whole.

Line 150: The authors mention that mosquito abundance was corrected for sampling effort when it was reported. What happened with the data for the studies that did not gave sampling effort, were they excluded? If not, how were they integrated? Perhaps using a negative binomial distribution with a zero-inflated correction would be better.

Figure 3: I do not believe an abundance in the negative is possible, please change scale
