# Supplementary material for: Differing taxonomic responses of mosquito vectors to anthropogenic land-use change in Latin America and the Caribbean
Source: PLoS Negl Trop Dis. 2023 Jul 14;17(7):e0011450. doi: 10.1371/journal.pntd.0011450 (PMC10348580; doi:10.1371/journal.pntd.0011450)
Supplement: Attachment — Submitted filename: PLOS NTD reviewer response March 23.docx [file pntd.0011450.s028.docx]

Response to editor and reviewer comments

PLOS Neglected Tropical Diseases submission PNTD-D-22-01333

*Differing taxonomic responses of mosquito vectors to anthropogenic land-use change in Latin America and the Caribbean*

We wish to thank the editors and reviewers for their valuable and kind comments on our manuscript, particularly their interesting comments and food for thought. This has led to several improvements to the manuscript including clarifications of methodology. In particular, we have emphasised our results demonstrating the strong impact of recent deforestation on *An. darlingi* and *An. albitarsis* abundance:

- Line 17: “*There has been a limited assessment of how mosquito vectors respond to land-use changes, including differential species responses, and the dynamic nature of these responses*.”
- Line 83: *“ However, there is limited understanding of whether mosquito responses to land use change are consistent at a regional scale. An assessment of how important vectors respond to land-use change and dynamic ecological alterations, such as deforestation, will be useful for designing control strategies that can be implemented at scale.*”
- Line 448: “*Overall, we found inconsistent spatial responses of mosquito biodiversity to land use in Latin America and the Caribbean, but a strong temporal signal to recent deforestation*.”
- Line 453: “*Further, we found a strong and consistent temporal signal of deforestation on both dominant and secondary malaria vectors, highlighting the importance of considering the temporal dynamics of land use change in driving disease risk*.”
- Line 502: “*Here, An. darlingi exhibited the strongest response to recent deforestation, however there was wide uncertainty in the spatial effects of land use. This result is supported by previous findings demonstrating that An. darlingi is favoured by deforestation (12,13). Secondary growth, particularly at forest fringes created during the earlier and more rapid stages of deforestation, offers a range of suitable environmental conditions for An. darlingi, including increased sunlight, refugia and ground pools (15,65,66). Our detection of a stronger impact of the temporal effects of land use change, particularly deforestation on An. darlingi, in comparison to the uncertain spatial effects suggests that the dynamic nature of the land use change is highly important*.”
- We believe that highlighting this interesting finding more and comparing the strong temporal response of deforestation to the more uncertain spatial impacts of land use on mosquito biodiversity have improved the novelty of our manuscript.

**Reviewer 1**

*Firstly, we would like to thank the reviewer for their positive comments and taking the time to read our manuscript, we very much appreciate their comments. Please see below the responses to the individual comments.*

The main issue I have with the manuscript in it’s current form is related to the statistical analysis. The authors investigate land use and their respective effects on mosquito richness and abundance across 4 categories (primary vegetation, secondary vegetation, managed and urban), however, I believe that a more solid approach related to urban gradients and land-use would be to use a continuous variable rather than an assigned category. For instance, using the APPEARS resource on EarthData would allow the authors to use more adequate proxies of land-use, such as EVI or nightlight rasters, thus perhaps giving a different perspective on their results. This is but food for thoughts, as I believe the current analysis to have been done appropriately.

*We appreciate that our analysis could have been performed in a different way. However, since the data collection was conducted in line with other data collection efforts (the PREDICTS database), we believe that following similar statistical methodology was appropriate here. We acknowledge that in future, these data could indeed be combined with fine-scale satellite imagery to obtain estimates of land use.*

There is also a seemingly puzzling fact: looking at figure 2 and 3, there seem to be a decrease in both abundance and richness of mosquitoes in urban areas. Keeping in mind that these data were analysed as categories, I’m wondering why the authors found a positive abundance response to urbanisation in figure 4. I understand that figure 4 relates to changes in land use over time as opposed to assigned land-usage, but still this change in effect directionality seems unlikely. Again, this relates to my previous comment, and I’m wondering if using a continuous, satellite-based index would change the directionality of the effect shown in figure 2 and 3.

*Figure 4 shows the effect of deforestation only, and not urbanisation. Figure 4B shows that when deforestation is high, An. darlingi and An. albitarsis abundance is also high. This does not necessarily mean that these mosquitoes are favoured by urbanisation as deforestation does not always equal urbanisation. The deforestation data that we used for this part of our analysis was satellite-based (the Hansen data; see lines 240).*

Lastly, I have but a curiosity comment: since the authors are working on a multi-species system, would it be possible to create a phylogenetic tree of the mosquito species present in the collected database in order to include a phylogenetic signal in the analyses? I would not be too surprised to see recent evolutionary history playing a major role in shaping abundance and richness pattern linked with land-usage at a species level. Again, this is but food for thoughts.

*We thank the author for their food for thought and interesting discussion points brought up with this manuscript. As a potential next step, it would be possible to piece together a phylogenetic tree to examine evolutionary factors behind the mosquito responses. However, this was beyond the scope of the current study.*

Line 57: Global warming?

*We have changed to ‘climate change’*

Line 71: I am not sure this statement is true; I can think of a few studies that were published in the past few years specifically focused on large-scale mosquito patterns (see Steiger et al. 2016, Rakotoarinia et al. 2022), and even more so on the amazon basin (MacDonald and Mordecal 2019).

*This has been changed to “mosquito responses at a regional scale have not been well explored”.*

Line 78: I would argue that it negatively impacts native biodiversity, not biodiversity as a whole.

*This has been changed to “negatively impacts native terrestrial biodiversity…”*

Line 150: The authors mention that mosquito abundance was corrected for sampling effort when it was reported. What happened with the data for the studies that did not gave sampling effort, were they excluded? If not, how were they integrated? Perhaps using a negative binomial distribution with a zero-inflated correction would be better.

*We only included studies which reported sampling effort, and therefore we could include an effort-corrected abundance. We have made this clearer in our methods by editing line 173: “For studies where sampling effort varied across sample sites, raw species-level abundance measurements were divided by sampling effort to give an effort-corrected abundance measurement.” This is also detailed in the Supplementary Materials page 2 “Studies were also excluded if only one land-use type was sampled and if the sampling methodology was not adequately described, for example if the study did not include sampling effort*.*”*

Figure 3: I do not believe an abundance in the negative is possible, please change scale.

*The negative represents a reduction in abundance of the mosquitoes. i.e. looking at Aedes scapularis, abundance at primary vegetation-substantial sites was approximately 50% less than the abundance at our baseline (primary vegetation-minimal). This shows that Aedes scapularis is negatively impacted by this land use change.*

**Reviewer 2**

This study aims to characterize the effects of land-use changes on the abundance and diversity of mosquitoes. The approach is very interesting given the amount of data available. However, I find that the manuscript is very focused on one result: All mosquito species do not respond in the same way to land-use changes, while this result is already well known and synthesized in recent reviews. Conversely, a result that I found very interesting but not really highlighted and detailed in the manuscript is the effect of the dynamic of deforestation on the abundance of several species, such as *An. darlingi*. When deforestation is rapid/severe/high (in the 5 years before the sampling), species abundance increases more than when deforestation is slow/low. This is a surprising result because for the same species the response to land-use changes was not significant (no difference in abundance between urban and forest habitat). This therefore suggests that the dynamic of land-use change may be more important than the change itself in explaining the observed patterns. This conclusion should be further investigated and discussed in the manuscript because this is an important information for conservation biology and landscape management.

*We thank the reviewer for their comments and opportunity to improve our manuscript. We agree that the deforestation results are interesting and could be highlighted more. Therefore, we have made a number of additions and clarifications, particularly comparing the high uncertainty in the spatial impacts of land use change e.g. An. darlingi in Figure 3, with the strong temporal response of An. darlingi to deforestation in Figure 4B. This reflected in our manuscript:*

- Line 17: “*There has been a limited assessment of how mosquito vectors respond to land-use changes, including differential species responses, and the dynamic nature of these responses*.”
- Line 83: *“ However, there is limited understanding of whether mosquito responses to land use change are consistent at a regional scale. An assessment of how important vectors respond to land-use change and dynamic ecological alterations, such as deforestation, will be useful for designing control strategies that can be implemented at scale.*”
- Line 448: “*Overall, we found inconsistent spatial responses of mosquito biodiversity to land use in Latin America and the Caribbean, but a strong temporal signal to recent deforestation*.”
- Line 453: “*Further, we found a strong and consistent temporal signal of deforestation on both dominant and secondary malaria vectors, highlighting the importance of considering the temporal dynamics of land use change in driving disease risk*.”
- Line 502: “*Here, An. darlingi exhibited the strongest response to recent deforestation, however there was wide uncertainty in the spatial effects of land use. This result is supported by previous findings demonstrating that An. darlingi is favoured by deforestation (12,13). Secondary growth, particularly at forest fringes created during the earlier and more rapid stages of deforestation, offers a range of suitable environmental conditions for An. darlingi, including increased sunlight, refugia and ground pools (15,65,66). Our detection of a stronger impact of the temporal effects of land use change, particularly deforestation on An. darlingi, in comparison to the uncertain spatial effects suggests that the dynamic nature of the land use change is highly important*.”

L16: To avoid the repetition of 'including', I propose to replace 'including dengue and malaria' by '(e.g., dengue and malaria)'.

*Thank you, this has been changed.*

L30: I suggest to homogenize land use change/land-use change/land-use changes throughout the manuscript.

*This has been homogenised to ‘land-use change’.*

L150: Are there studies included in the analyzes whose sampling effort was not reported? If yes, how many? *No, we only included studies where sampling effort was recorded.*

*We have made this clearer in our methods by editing line 173: “For studies where sampling effort varied across sample sites, raw species-level abundance measurements were divided by sampling effort to give an effort-corrected abundance measurement.” This is also detailed in the Supplementary Materials page 2 “Studies were also excluded if only one land-use type was sampled and if the sampling methodology was not adequately described, for example if the study did not include sampling effort*.*”*

L167: How is the use of multiple capture methods within a study modeled?

*This was captured by assigning each record a ‘study sample’, i.e. if there were multiple capture methods then the study sample would be different for each abundance measurement. Study sample was then included as a random intercept term in the model, this is detailed in the following lines:*

*Line 142: “Specifically, each record was assigned a study number (a unique paper), site number (a geographic location at which mosquito abundance was sampled), study block (a collection of sites within a distinct spatial cluster, to account for spatial autocorrelation within a study) and study sample (a sample with consistent sampling methodology, such as capture method and sample month)*”

*Line 207: “Other random effects considered in the model structure included study block, used to account for spatial autocorrelation between sites within a study and study sample*.”

L169: ‘for each unique species at each site’ is not clear for me. In the supplementary materials, it is only a random effect ‘species’, in this case I suggest to remove ‘at each site’.

*This has been removed.*

L175-183: Should the results be given with all the random effects included in the models? The majority of them are included to take into account the non-independence of the data, whether they have a significant effect or not.

*We decided to include all the random effects due to the vast variation in sites, including sampling methodologies. We believe that this means when interpreting our results, we can assume the effect is mainly due to land-use change, rather than site and sampling differences.*

L272: Repetition (L190-191).

*We have removed the following from line 340 “where 12.5% of the data was held out at a time from the models”*

L311-312: I suggest to give this information for all species since it could be interesting for the reader to see the balance or imbalance of sampling among species and among land-use types (either in an additional figure/table or directly integrated into the figures of the manuscript).

*We have provided this information (for all species in Figure 3) in an additional Table S15.*

L318: ‘In contrast to spatial responses of mosquito biodiversity’. The following results do not contrast with the previous result, they are rather in agreement: the first shows a decline in mosquito richness and abundance in urban areas, and the second shows a negative effect of deforestation.

*This has been removed.*

L318-331: Is it really temporal? Here, several sites with different levels of deforestation in the last 5 years are compared. For a temporal approach, several records are needed within the same sampling site over time and linked to deforestation which acts at the same time. Rather than temporal deforestation, I would use deforestation intensity, severity, rapidity or something like that. In fact, the Figure 4 showed that the greater the deforestation in previous years following the sampling, the greater the abundance and richness of mosquitoes. Therefore, 'fast' deforestation does not have the same effect as 'slow' deforestation. This is an original and interesting result.

*We have removed this word (line 410).* *The use of the word temporal here is in reference to the contrasting effect between the first part of the analysis, where we examine the impact of different land use types at different sites on mosquito biodiversity. In contrast, the deforestation analysis represents the impact of deforestation at a set point in time i.e. recent and rapid deforestation. In future analyses, we could explore the impact of longer term deforestation by using estimates of forest loss over five years. Regarding this interesting result, we have emphasised this more in our manuscript.*

L479-513: These three paragraphs deal with the importance of considering species-specific responses to land-use changes, but this result is already known in the literature. Could the results presented in this study allow us to go further? If so, I would rather discuss that, if not, I would synthesize these three paragraphs.

*We have cut down lines 396-413 to:*

*“In addition to genus-specific responses to land-use change, we found pronounced species-specific abundance responses, highlighting the complexity in generalising mosquito responses to understand disease risk. The uncertainty in mosquito responses to land use also suggests that spatial effects are highly variable over a large geographical area. The differential response of mosquito species to land-use change is likely to be driven by the unique life-history characteristics and habitat preferences of each species (54,55). We found increased abundance of opportunistic species in disturbed landscapes, including Ae. aegypti and Ae. albopictus mosquitoes. Human-dominated landscapes provide a range of novel habitats that facilitate increased abundance, densities, development and survival of Aedes mosquitoes in urban and agricultural areas (56,57). These findings have implications for the emergence of arboviruses transmitted by Ae. aegypti and Ae. albopictus, such as dengue, yellow fever and chikungunya, as disease transmission could be facilitated in anthropogenic environments without the implementation of adequate control measures.”*

L403: More importantly, it is not the deforestation but the intensity of forest loss in the years before the sampling that seems to affect its abundance (the dynamic of deforestation is therefore highly important).

*We have added lines 507-510 to highlight the importance of the dynamic nature of deforestation:*

*“Our detection of a stronger impact of the temporal effects of land-use change, particularly deforestation on An. darlingi, in comparison to the uncertain spatial effects suggests that the dynamic nature of the land-use change is highly important.”*

**Reviewer 3**

what do you mean "at a time" (need to clarify) (line 191)

*By this, we mean that random subsampling was performed by excluding the first 12.5% of the data, the following 12.5% and so on until eight subsamples of the data had been excluded.*

*Line 227: “Finally, for the random subsampling, eight hold-out models were fitted excluding 12.5% samples of the data at a time.”*

Although Brazil is mentioned as one of the countries included in your article, other countries were not mentioned in the results. It'll be useful to name the countries and the number of studies so the reader get better idea of the proportion of studies per country and maybe type of site. My suggestion is to include a table in the results section with this information (ex. country, number of studies used, type of site/ecosystem (urban, tropical rain forest, tropical dry forest, etc.), and author citation). It could serve as a quick reference for the reader. I still like the map and I think it still could be used.

*We make particular reference to Brazil because most of the data (67%) were from this country. We have named other countries in the text (line 262) and have added a table (Table S10) with a breakdown of sites by country.*

Addition of p-values to results

*Since we used Bayesian statistical analysis, p values are not obtained. Instead, we have provided 95% credible intervals e.g. line 295.*

Why do you have 94% and 95% for primary and 68% for secondary. the two values for 1ry do not make sense (shouldn't it be only one %?) (lines 329)

*As mentioned above, the 95% is in reference to the 95% credible intervals associated with Bayesian statistics and the number before represents the 94% increase/decrease abundance with land use.*
