# Supplementary material for: Differing taxonomic responses of mosquito vectors to anthropogenic land-use change in Latin America and the Caribbean
Source: PLoS Negl Trop Dis. 2023 Jul 14;17(7):e0011450. doi: 10.1371/journal.pntd.0011450 (PMC10348580; doi:10.1371/journal.pntd.0011450)
Supplement: Attachment — Submitted filename: Reviewer response - IFletcher.docx [file pntd.0011450.s030.docx]

Response to reviewer comments

PLOS Neglected Tropical Diseases submission PNTD-D-22-01333

*Differing taxonomic responses of mosquito vectors to anthropogenic land-use change in Latin America and the Caribbean*

*We wish to thank all the reviewers for their positive feedback regarding the revisions made to the manuscript and for acknowledging our efforts in addressing their comments. We appreciate their thorough review of our paper and have addressed the remaining comments below.*

Reviewer #2: L178-180 ‘The best-fitting random effects structure was selected by formulating iterative models of each response (i.e. abundance and richness) with the addition of each random effect (Table S7-8)’ & L183-184 ‘Random effects were retained in the final model structure if the model was improved.’
Why are random effects tested? As they are there to take into account the non-independence of the data, they should not be removed from the model. Are the results the same when all random effects are included (even those that do not improve the fit of the models)?

*The random effects were indeed included in our models to take into account extra dependencies in the data and to address additional sources of variation (variability between studies, study sites, species, study blocks, and ecoregions). However, it is possible that including certain random effects may not substantially improve model fit or contribute to explaining variation. Instead, including some random effects may add extra complexity to our models (assessed through a change in DIC and WAIC). Based on our testing of the random effects our results are consistent, which is why we chose to include all the random effects in our final models.*


Reviewer #3: THERE ARE FEW GRAMMATICAL ERRORS THAT NEED ATTENTION.
*Thank you for bringing these to our attention, we have now corrected these grammatical errors.*
